# Are ecological communities the seat of endosymbiont horizontal transfer and diversification? A case study with soil arthropod community

**DOI:** 10.1002/ece3.8108

**Published:** 2021-10-16

**Authors:** Manisha Gupta, Rajbir Kaur, Ankita Gupta, Rhitoban Raychoudhury

**Affiliations:** ^1^ Indian Institute of Science Education and Research Mohali (IISER‐Mohali) Manauli India; ^2^ Indian Institute of Science Bengaluru India; ^3^ ICAR‐ National Bureau of Agricultural Insect Resources (NBAIR) Bengaluru India

**Keywords:** DNA barcoding, ecological community, endosymbionts, horizontal transfer, recombination

## Abstract

Maternally inherited endosymbionts of arthropods are one of the most abundant and diverse group of bacteria. These bacterial endosymbionts also show extensive horizontal transfer to taxonomically unrelated hosts and widespread recombination in their genomes. Such horizontal transfers can be enhanced when different arthropod hosts come in contact like in an ecological community. Higher rates of horizontal transfer can also increase the probability of recombination between endosymbionts, as they now share the same host cytoplasm. However, reports of community‐wide endosymbiont data are rare as most studies choose few host taxa and specific ecological interactions among the hosts. To better understand endosymbiont spread within host populations, we investigated the incidence, diversity, extent of horizontal transfer, and recombination of three endosymbionts (*Wolbachia*, *Cardinium,* and *Arsenophonus*) in a specific soil arthropod community. *Wolbachia* strains were characterized with MLST genes whereas 16*S rRNA* gene was used for *Cardinium* and *Arsenophonus*. Among 3,509 individual host arthropods, belonging to 390 morphospecies, 12.05% were infected with *Wolbachia*, 2.82% with *Cardinium* and 2.05% with *Arsenophonus*. Phylogenetic incongruence between host and endosymbiont indicated extensive horizontal transfer of endosymbionts within this community. Three cases of recombination between *Wolbachia* supergroups and eight incidences of within‐supergroup recombination were also found. Statistical tests of similarity indicated supergroup A *Wolbachia* and *Cardinium* show a pattern consistent with extensive horizontal transfer within the community but not for supergroup B *Wolbachia* and *Arsenophonus*. We highlight the importance of extensive community‐wide studies for a better understanding of the spread of endosymbionts across global arthropod communities.

## Graphical abstract

This manuscript characterizes incidence, diversity, extent of horizontal transfer, and recombination of three different endosymbionts (*Wolbachia*, *Cardinium*, and *Arsenophonus*) in a soil arthropod community. We utilized multilocus strain typing system (MLST) for *Wolbachia* and 16S rRNA gene for *Cardinium* and *Arsenophonus* to answer whether endosymbionts are being transferred within an ecological community first and then spreading through different arthropod communities across the world.

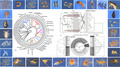

## 1 INTRODUCTION

Maternally inherited endosymbionts, infecting arthropods, are one of the most diverse and abundant of all bacteria infecting them. About two‐third of all terrestrial arthropods are infected with at least one maternally inherited endosymbiont (Hilgenboecker et al., 2008) and can play crucial roles in the ecology and evolution of their hosts (Gebiola et al., 2017; Semiatizki et al., 2020). The most common of these endosymbionts are *Wolbachia, Cardinium, Arsenophonus, Rickettsia* and *Spiroplama*. Out of these, *Wolbachia* remains the most widely distributed with an incidence rate of 16–66% (Hilgenboecker et al., 2008; Weinert et al., 2015; Werren, Windsor, et al., 1995) and comprising of 18 different clades (supergroups A to R) (Landmann, 2019). Incidence of the other endosymbionts varies from 4–10% (Duron et al., 2008; Zchori‐Fein & Perlman, 2004).

An important evolutionary feature of these endosymbionts is the lack of phylogenetic congruence with their hosts (Dewayne Shoemaker et al., 2002; Werren, et al., 1995). This indicates that although they undergo vertical transmission in the short term, over the course of their evolutionary histories, they have also undergone extensive horizontal transfer across taxonomically unrelated hosts (Werren et al., 2008). This is also evident from the occurrence of similar endosymbiont strains in taxonomically unrelated hosts and conversely, the presence of divergent strains in closely related hosts (Vavre et al., 1999). Individual arthropods can harbor a single or multiple strain of any one endosymbiont as well as multiple strains of different endosymbionts (Zhang et al., 2016). The presence of more than one type of endosymbiont in a single host can be indicative of the utilization of the same host by different endosymbionts to spread across different arthropod communities (Russell et al., 2012; Zélé et al., 2018; Zhao et al., 2013).

Another key feature of endosymbionts is the pervasive recombination seen in their genomes (Ellegaard et al., 2013). This has been particularly well documented in *Wolbachia* (Malloch & Fenton, 2005) as well as in other endosymbionts (Mouton et al., 2012). This level of recombination is extensive enough to render single‐gene phylogenies unable to properly reflect the evolutionary history of an endosymbiont strain and, therefore, has necessitated the development of multilocus strain typing (MLST) systems (Maiden et al., 1998). Such MLST studies (Hou et al., 2020), as well as whole‐genome analysis of endosymbionts (Wang et al., 2020), indicate extensive recombination within them. However, for recombination to happen, at least two endosymbiont strains must be present in the same cytoplasm of a particular host. This is possible through horizontal transfer of endosymbionts to different hosts. During this process, different endosymbionts can find themselves sharing the same cytoplasm with resident endosymbionts and can also lead to multiple infections. The rate at which this leads to stable multiple infections is not known, but this obviously creates opportunities for genetic exchange between endosymbionts. Moreover, such coinfections can trigger selection whereby only a single endosymbiont can remain within a host. Such flux seems to be a key feature of endosymbiont dynamics, especially with *Wolbachia*, where loss is 1.5 times higher than acquisition of new infections (Bailly‐Bechet et al., 2017). Additionally, this co‐occurrence can lead to increased chances of recombination between these strains especially if the new host is already infected with other strains. Evidence for such recombination is also well documented. The presence of a very similar recombinant *Wolbachia* strains has been observed in the parasitoid wasp *Nasonia* and its host *Protocalliphora* (Werren & Bartos, 2001), as well as in *Anastrepha* fruit flies and their parasitoid braconid wasps (Mascarenhas et al., 2016).

Horizontal transfer, therefore, can not only increase the taxonomic range of hosts that a particular endosymbiont can infect, but can also be responsible for increased incidences of recombination between endosymbionts. Moreover, horizontal transfer of endosymbionts can increase if different hosts, with different resident infections, come in contact like in host–parasite, host–parasitoid, prey–predator, and other ecological interactions (Sanaei et al., 2021). Examples where host–parasitoid interactions have been implicated for such transfer include the presence of similar *Wolbachia* strains among frugivorous *Drosophila* and their hymenopteran parasitoid (Vavre et al., 1999), *Nasonia vitripennis* and *Muscidifurax uniraptor* sharing similar *Wolbachia* with their fly host *Protocalliphora* (Baudry et al., 2003) and transmission of *Wolbachia* into whitefly via parasitoid wasps (Ahmed et al., 2016). Another such ecological association which can lead to endosymbiont transfer is prey–predator interactions such as the predatory mite *Metaseiulus occidentalis* and its prey *Tetranychus urticae* (spider mite) sharing similar endosymbionts (Hoy & Jeyaprakash, 2005). Some parasitic mites, such as *Tyrophagus putrescentiae*, can also facilitate the transfer of *Wolbachia* to different *Drosophila* host populations (Brown & Lloyd, 2015). Similarly, horizontal transfer of endosymbionts can also be host plant mediated as observed in the transfer of *Cardinium* to different leafhopper species (Gonella et al., 2015) as well as horizontal transfer of *Wolbachia* in whitefly through cotton leaves (Li et al., 2017). Shared food resources between hosts can also mediate horizontal transfer of endosymbionts (Tolley et al., 2019).

It is clear from these examples that horizontal transfer of endosymbionts is possibly taking place when two hosts are coming together to perform a particular ecological interaction and thus have overlapping niches (Sanaei et al., 2021). The endosymbiont present within these hosts can then be serendipitously transferred from one host to the other. Therefore, to understand the dynamics of the spread of endosymbionts through horizontal transfer, one needs to look at the level where most of these ecological associations are taking place, which could be within the boundaries of ecological community. A well‐defined ecological community will have several diverse host taxa with significant overlap of niches as they are interacting with each other. This physical contact of the hosts can facilitate the horizontal transfer of their resident endosymbionts (Sanaei et al., 2021). Moreover, many host taxa can belong to many different ecological communities (Morrow et al., 2014) and this cosmopolitan nature of a few host taxa will further facilitate the spread of endosymbionts from one ecological community to another, almost like spreading through a metacommunity (Brown et al., 2020). Therefore, investigating endosymbiont diversity and horizontal transfer within specific ecological communities seems logical. Yet, there are very few studies that have taken this approach and instead focus mainly on endosymbiont spread within a particular habitat (Stahlhut et al., 2010) or in a specific genus (Baldo et al., 2008; Raychoudhury et al., 2009; Turelli et al., 2018) or within specific host taxa (Ahmed et al., 2016; Tolley et al., 2019). Among community‐wide surveys, Kittayapong et al. (2003) demonstrated *Wolbachia* strain diversity within rice field arthropod community, whereas Sintupachee et al. (2006) reported plant‐mediated horizontal transfer among arthropod community found on pumpkin leaves. However, to understand whether such horizontal transfer can be correlated with relatively higher rates of recombination is difficult as most of these studies are based on single‐gene phylogenies. Another crucial effect of this view of within‐community horizontal transfer of endosymbionts can lead to an important hypothesis about sequence diversity of the endosymbionts themselves. If endosymbionts are rapidly undergoing horizontal transfer within a particular ecological community, then very similar bacterial strains would be found among the arthropod hosts of that community. This would make these bacteria more closely related to each other, resulting in lower than expected pairwise sequence divergence among them. This can then serve as a signature of recent and relatively rapid community‐wide horizontal transfer of resident endosymbionts.

In the present study, we test whether such horizontal transfer and resulting recombination are happening within the endosymbionts of a diverse soil arthropod community. Three major endosymbionts, *Wolbachia, Cardinium,* and *Arsenophonus*, were selected and screened across arthropod hosts. We investigated *Wolbachia* sequence diversity using the well‐established MLST scheme (Baldo et al., 2006) and also identified specific recombination events. We also investigated *Cardinium* and *Arsenophonus* incidence with 16*S rRNA* gene sequences. A statistical model was then used to test whether the endosymbiont found within this community is more closely related to each other than expected. Our study indicates that supergroup A *Wolbachia* and *Cardinium* are indeed showing greater sequence similarity within the community indicating, perhaps, that such horizontal transfer events are more prevalent in these two endosymbionts than in supergroup B *Wolbachia* and *Arsenophonus*.

## 2 MATERIALS AND METHODS

### 2.1 Sample collection and morphological identification of soil arthropods

To access soil arthropod biodiversity, sampling was done in October and November 2015 (a postmonsoon season) from a relatively undisturbed land (220 × 70 m^2^) near the vicinity of the host institution (30°39′N 76°43′E, Mohali, Punjab, India; Figure S1A). The study area was naturally divided into roughly eight quadrants by plantations of poplar (Figure S1B,C). Five randomly selected quadrants were sampled by collecting leaf litter and pitfall traps. These two independent sampling methods were used in tandem to give a more comprehensive sampling of the resident species (Olson, 1991; Querner & Bruckner, 2010) as pitfall traps are biased toward surface‐active taxa, whereas leaf litter method is biased toward less active taxa. Two parallel transects, each 30 m long, were marked across each quadrant using a rope (Figure S1B,D). Each of these transects was marked at 10 m intervals, and two alternatively marked points were sampled for leaf litter, while pitfall traps were placed at the other two ends (Figure S1E). Samples from each type of collections were later combined. In total, 20 collections, each from leaf litter and pitfalls, were obtained.

Leaf litter was collected from an area of roughly 0.09 m^2^ (Figure S1F) and immediately placed in a plastic bag. Additionally, each leaf litter sample was accompanied by a soil sample of an area ~282 cm^3^ immediately below it (Sabu & Shiju, 2010). Samples were weighed so that each sample roughly had the same weight (500–600 gm) and were then settled in a series of Tullgren funnels with a 100 W light. The emerging arthropods were collected in a 50 ml beaker, with absolute ethanol, continuously for the next 4–6 days or until no arthropod samples emerged. Emerged arthropods were collected every 24 hr, and 100 ml of fresh absolute ethanol was added to the collection beaker. The pitfall traps were settled on the ground by placing a 250‐ml beakers with 50 ml of absolute ethanol (Figure S1G). Samples were collected every other day with a replacement of fresh ethanol.

Arthropods obtained from each of pitfall and leaf litter samples (20 samples each) were sorted individually with multiple washes in fresh ethanol to reduce cross‐contamination. Detailed (dorsal and lateral) views of each individual arthropod obtained were photographed under a stereomicroscope (M205C, Leica Microsystems) with scale ranging from 0.2 to 2 mm. These were then sorted according to their morphology into morphospecies and provisionally identified till order level. To evaluate whether the sampling method employed yielded a significant proportion of the community diversity, several diversity indices were calculated with EstimateS v9.1.0 (Colwell, 2013) such as ACE (Abundance coverage estimator; Chao et al., 2000), Chao1 (Chao, 1984), ICE (Incidence coverage estimator), Chao2, and Jack 1 and Jack 2 (Smith & van Belle, 1984).

Cross‐contamination of both host and endosymbiont DNA is one of the possible caveats (Zettler et al., 2020), of collecting arthropod samples from the environment in bulk and subsequent storage. Although, previous reports indicate that this is unlikely (Duplouy et al., 2009), yet to minimize this, the collected and sorted morphospecies were washed in ethanol multiple times.

For morphospecies which had more than three individuals, DNA was extracted from a single individual by either the HiPurA™ insect DNA purification kit (HIMEDIA) or by using the phenol–chloroform–isoamyl alcohol (PCI) method. In PCI method, samples were crushed in 200 µl lysis buffer containing 10 mM each of Tris‐HCl (pH 8.0), EDTA (pH 8.0) and NaCl. DNA was precipitated using isopropanol and dissolved in 1X TE (pH 8.0). For morphospecies which only had single individuals, a different nondestructive extraction protocol was used (Rowley et al., 2007). Whole individuals were incubated at 60℃ in 100‐400 µl of guanidinium thiocyanate (GuSCN) based extraction buffer (GuSCN, 0.1M Tris‐HCl, 0.2M EDTA with Triton X‐100) for 1–4 hr. The individuals were then removed for storage, and the DNA remaining in the buffer was precipitated using isopropanol. Extracted DNA was quantified using the NanoDrop™ 2000 spectrophotometer (Thermo Fisher Scientific), and PCR suitability was accessed by running a PCR using 28*S* primers (Table 1).

**TABLE 1:** Primers used in this study

	Gene	Primer	5′−3′	Amplicon size	Annealing temperature (°C)	Reference
Arthropod host	28*S*	28s_F	CCCTGTTGAGCTTGACTCTAGTCTGGC	500	55	Werren, Windsor, et al. (1995)
		28s_R	AAGAGCCGACATCGAAGGATC			
	*CO1*	LCO1490	F‐GGTCAACAAATCATAAAGATATTGG	700	55	Vrijenhoek (1994)
		HC02198	R‐TAAACTTCAGGGTGACCAAAAAATCA			
		LEP‐F1	ATTCAACCAATCATAAAGATAT	600	55	Hebert et al. (2003)
		LEP‐R1	TAAACTTCTGGATGTCCAAAAA			
*Wolbachia*	16*S*	*wspec* F	CATACCTATTCGAAGGGATAG	500	55	Werren and Windsor (2000)
		*wspec* R	AGCTTCGAGTGAAACCAATTC			
	*gatB*	*gatB* general	F‐GAKTTAAAYCGYGCAGGBGTT	369	52	Baldo et al. (2006)
			R‐TGGYAAYTCRGGYAAAGATGA			
		*gatB* A specific	F‐TTTAGAGCAAGATGCAGGRAAGAGCG		57.5	
			R‐TGGYAAYTCRGGYAAAGATGA			
		*gatB* B specific	F‐TAAGAATCGCAAGAATTCAC		57.5	
			R‐TGGYAAYTCRGGYAAAGATGA			
	*coxA*	*coxA* general	F‐TTGGRGCRATYAACTTTATAG	402	51	
			R‐CTAAAGACTTTKACRCCAGT			
		*coxA* A specific	F‐ATACCCACCTTTATCACAGG		59	
			R‐CTAAAGACTTTKACRCCAGT			
		*coxA* B specific	F‐ATACCCACCTYTRTCGCAAA		57	
			R‐CTAAAGACTTTKACRCCAGT			
*Wolbachia*—MLST genes	*hcpA*	*hcpA* general	F‐GAAATARCAGTTGCTGCAAA	444	51	
			R‐GAAAGTYRAGCAAGYTCTG			
		*hcpA* A specific	F‐GAAATARCAGTTGCTGCAAA		56	
			R‐TTCTARYTCTTCAACCAATGC			
		*hcpA* B specific	F‐GAAATARCAGTTGCTGCAAA		56	
			R‐TTCTTTGTCGCTMACTTYAATCAKG			
	*ftsZ*	*ftsZ* general	F‐ATYATGGARCATATAAARGATAG	435	54	
			R‐TCRAGYAATGGATTRGATAT			
		*ftsZ* A specific	F‐AAAGATAGTCATATGCTTTTC		53	
			R‐CATCGCTTTGCCCATCTCG			
		*ftsZ* B specific	F‐AAAGATAGCCATATGCTCTTT		55	
			R‐CATTGCTTTACCCATCTCA			
	*fbpA*	*fbpA* general	F‐GCTGCTCCRCTTGGYWTGAT	429	56	
			R‐CCRCCAGARAAAAYYACTATTC			
		*fbpA* A specific	F‐GTTAACCCTGATGCTTATGAC			
			R‐CCRCCAGARAAAAYYACTATTC			
		*fbpA* B specific	F‐GTTAACCCTGATGCTTACGAT			
			R‐CCRCCAGARAAAAYYACTATTC			
*Cardinium*	16*S*	Clo‐f	F‐GGAACCTTACCTGGCCTAGAATGTATT	500	56	Gotoh et al. (2007)
		Clo‐r	R‐GCCACTGTCTTCAAGCTCTACCAAC			
*Arsenophonus*	16*S*	Ars‐F	F‐GGGTTGTAAAGTACTTTCAGTCGT	600	55	Duron et al. (2008)
		Ars‐R3	F‐CCTYTATCTCTAAAGGMTTCGCTGGATG			

The morphospecies were barcoded (Hebert et al., 2003) using the ~600 bp of the mitochondrial *CO1* gene (Table 1). 2–20 ng/µl of extracted DNA was used in 20 µl PCRs with an initial denaturation step at 95℃ for 3 min, 39 cycles of denaturation (95℃, 45 s), annealing (51–56℃ for 45 s), extension (72℃, 1 min), and a final extension at 72℃ for 10 min. PCR products were visualized on 1% agarose gels and then cleaned with Exonuclease I and Shrimp alkaline Phosphatase (New England Biolabs Inc.) before being sequenced with the BigDye^®^ Terminator v3.1 Cycle Sequencing Kit. Initially, only the forward strand was sequenced and if any base ambiguity was observed, then the reverse strand was also sequenced. This was especially done to detect any cases where due to prey–predator, host–parasite, host–parasitoid, and other ecological interactions, multiple arthropod hosts could have been present in any one sample. These hosts would then yield multiple peaks in the *CO1* chromatograms as DNA from more than one host would be coamplified. However, we did not find any such discrepancy in our dataset.

*CO1* sequences obtained were identified through the NCBI (Johnson et al., 2008) and BOLD databases (Ratnasingham & Hebert, 2007) by BLAST (last performed in August 2019). The “best hit” obtained was used to check the provisionally identified morphospecies. If both databases yielded the same hit, then it was determined to have been identified. If they yielded different hits, then the taxonomic identification was moved down to the level common between these two hits. These results were further cross‐checked with the photographic data to establish whether the *CO1* sequences also matched up with the provisional taxonomic identity of the morphospecies. Only unique morphospecies were included in further analysis after analyzing the *CO1* sequences (Table S2).

### 2.2 Endosymbiont screening and phylogenetic analysis

All the 390 morphospecies obtained were screened for the three endosymbionts*—Wolbachia*, *Cardinium,* and *Arsenophonus*. Incidence of each of these endosymbionts was estimated using primers specific to them (Table 1). The multilocus strain typing (MLST) system was used (Baldo et al., 2006) to identify and characterize the *Wolbachia* infections. For *Cardinium* and *Arsenophonus,* 16*S rRNA* gene was amplified using specific primers (Table 1). *Nasonia vitripennis* infected with both A and B supergroups of *Wolbachia* was used as positive control for *Wolbachia* screening, and *Bemisia tabaci* strains with *Cardinium* and *Arsenophonus* infections were used for screening the other two endosymbionts. Autoclaved distilled water was used as no‐template negative controls.

To test for the presence of *Wolbachia*, the *wspec* primers were used (Table 1). Samples positive for *wspec* were then sequenced for one of the MLST genes, usually *fbpA*, to identify single *Wolbachia* infections by inspecting the chromatograms for multiple peaks. Samples with multiple *Wolbachia* infection were not processed further as assigning a particular *Wolbachia* MLST allele to a particular strain would have been impossible. The MLST allele sequences were compared with existing sequences in PubMLST database (Jolley et al., 2018) to identify their allele profiles (unique numbers assigned for each unique sequence) and sequence type (ST) which is a new strain type as defined by the combination of five MLST allele profiles (Baldo et al., 2006). Unique sequences were submitted to the PubMLST database for curation. Sequences obtained from this study were also deposited in NCBI and BOLD databases (Table S2).

Sequences were aligned with Sequencher 5.2.4 (Gene Codes Corporation) and manually edited with BioEdit v. 7.2.5 (Hall, 1999). DNA sequence evolution models were computed using MEGA7 (Kumar et al., 2016). GTR+g (general time‐reversible model with γ‐distributed rate variation) was found to be the best model for all *CO1* phylogenetic trees. The appropriate models found for other datasets were as follows: K2+g (Kimura's 2‐parameter model) for both *Cardinium* and *Arsenophonus*; T92+g+i (Tamura's 3‐parameter model with γ‐distributed rate variation and proportion of invariable sites) for concatenated MLST dataset; T92+g for *gatB*, *hcpA*, *ftsZ*, *fbpA* gene; HKY+g (Hasegawa, Kishino, and Yano's model) for *coxA*. Bayesian phylogeny was constructed for *CO1* sequences using MrBayes v3.2.5 (Ronquist et al., 2012). Each phylogenetic analysis was run at least twice and was accepted only if there was no change in the major branching order (Figure S2). Maximum‐likelihood phylogenetic trees of *Wolbachia, Cardinium, and Arsenophonus* were constructed in MEGA7 with 1,000 bootstrap replicates for each. Phylogenetic trees were visualized and edited with Figtree v1.4.2 (Rambaut, 2009).

To account for the frequent recombination seen in *Wolbachia* genomes, ClonalFrame v2.1 (Didelot & Falush, 2007) was used to infer phylogeny from MLST data. ClonalFrame was run for 3 × 10^5^ iterations with the first 50% iterations discarded as burn‐ins. Estimates of recombination rate were also obtained.

### 2.3 Identifying horizontal transfers, recombination events, and test of endosymbiont similarity

To test for horizontal transmission of endosymbionts across the soil arthropod hosts, three sets of analyses were done. The first was a qualitative estimation of the horizontal transfer obtained by comparing host and endosymbiont phylogenies. The second was a quantitative estimation of the correlation between the pairwise distance matrices of hosts and their corresponding endosymbionts by the Spearman method (*r*) of Mantel test for correlation (Legendre & Legendre, 2012) which was done in R v1.2.5. A total of five different correlations were computed with their corresponding host sequences: all the *Wolbachia*‐infected samples (33), only A supergroup *Wolbachia*‐infected samples (16), only B supergroup *Wolbachia*‐infected samples (15), only *Cardinium*‐infected samples (eight), and *Arsenophonus‐*infected samples (seven). In addition, ParaFit (Legendre et al., 2002) and PACo (Procrustean Approach to Cophylogeny) programs (Balbuena et al., 2013) were used to test for association between host and endosymbiont pairwise distances.

To identify and visualize recombination events within the *Wolbachia* concatenated MLST sequences (2079 bp), SplitsTree v4.14.8 (Huson et al., 2008) was used to construct phylogenetic network using uncorrected *p*‐distance and neighbor‐net method (Bryant & Moulton, 2004). To statistically evaluate presence of recombination, *Φ* test (Bruen et al., 2006) was used in SplitsTree v4.14.8. These identified events were then further evaluated in RDP4 v4.97 (Martin et al., 2015). This program has been used to detect recombination events in similar studies (Baldo, Bordenstein, et al., 2006; Hou et al., 2020; Yang et al., 2013) and uses a pairwise scanning approach to detect recombination within a group of aligned sequences (Martin et al., 2015; Martin & Rybicki, 2000). It uses several suits of programs such as RDP (Martin & Rybicki, 2000), GENECONV (Padidam et al., 1999), MaxChi (Smith, 1992), SiScan (Gibbs et al., 2000), BootScan (Martin et al., 2005), Chimera (Posada & Crandall, 2001), and 3Seq (Boni et al., 2007) to detect and identify recombination events. A recombination event was considered for further analysis only when it was shown to be statistically significant by at least three or more of these detection methods. RDP was also used to detect the putative recombination breakpoints and their approximate positions in the concatenated dataset.

The model from Baldo et al. (2008) was used to test for the similarity of endosymbionts, where pairwise distance of endosymbiont strains found within the community was compared with strains available from different databases. To compare *Wolbachia*, MLST sequences, in the form of *Wolbachia* STs, were obtained from PubMLST database (last checked May, 2019). These were put in phylogenetic analyses with known supergroup sequences to identify whether they belonged to the A or the B supergroup. For *Cardinium* and *Arsenophonus,* homologues were obtained through NCBI Blast search and only those sequences were retained which had the same region of 16*S rRNA* amplified (last checked May, 2020). Mean pairwise distances were calculated in MEGA7 (Kumar et al., 2016) and then corrected with Jukes and Cantor model in DNAsp v5.10.01 (Librado & Rozas, 2009). Mean pairwise distances were computed for endosymbionts within the community, their expected value obtained from the equivalent number of pairwise distances randomly selected from the database and all unique endosymbiont sequences obtained from PubMLST and NCBI databases. Pairwise distances were also computed separately for *Wolbachia* supergroup A, supergroup B, *Cardinium,* and *Arsenophonus*. The mean pairwise distance of endosymbionts presents in soil arthropod community was compared with an equivalent number of pairwise distances randomly selected from the database for 10,000 iterations. These iterations were computed to give a null distribution for comparison with the soil endosymbiont sequence data by Wilcox rank‐sum test with continuity correction and 95% confidence interval (performed in R v1.2.5). Density plots for endosymbiont divergence from the soil arthropods and the databases were plotted in R (http://R‐project.org).

## 3 RESULTS

### 3.1 Morphospecies diversity and endosymbiont infection frequencies

**FIGURE 1:**
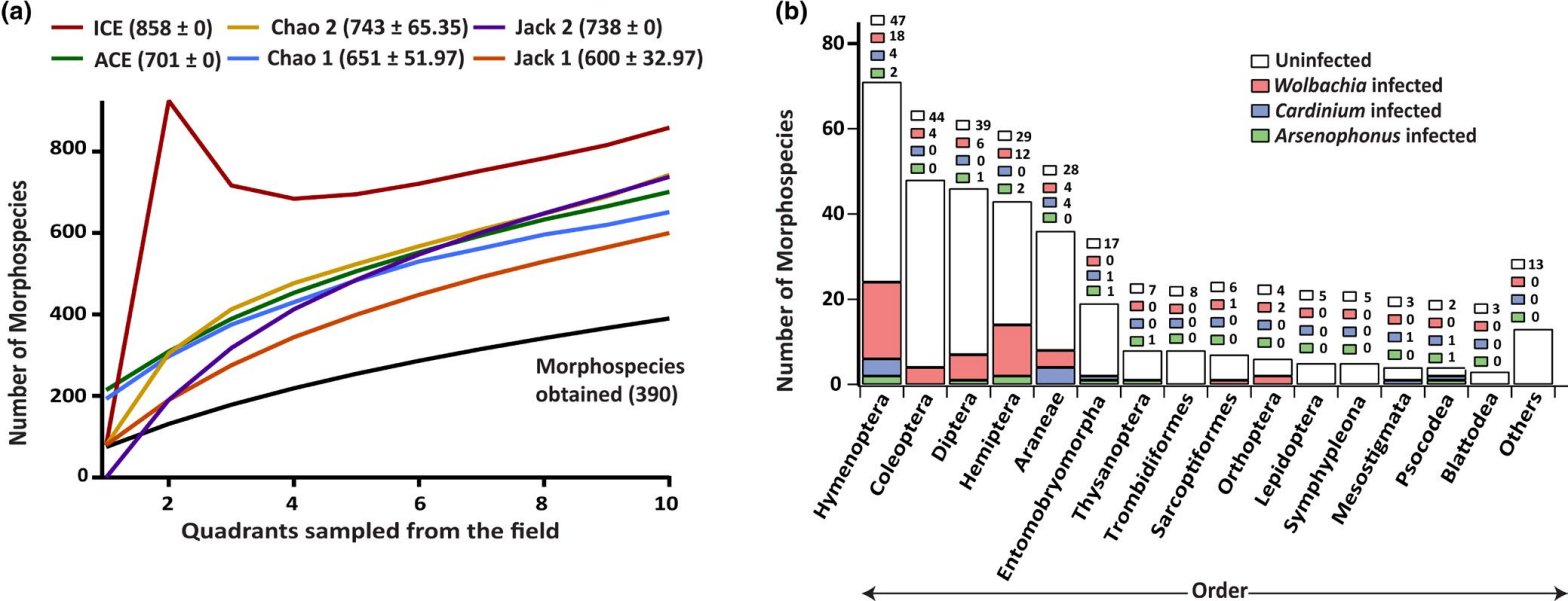
(a) Rarefaction curve of morphospecies found (in black) showing species richness in the soil arthropod community. Colored lines represent expected number of morphospecies. (b) The distribution of three endosymbionts screened across different host arthropod orders

A total of 3,509 individual arthropods were collected and sorted into 390 different morphospecies. Out of these, 198 morphospecies were exclusively obtained from the pitfall traps, 123 were obtained from leaf litter sampling, and 69 morphospecies were obtained from both. EstimateS provided diversity estimates ranging from 858 (±0), obtained from incidence coverage estimator, to 600 (±32.97) obtained through Jack 1 (Table S1). This indicates our sampling could capture 45–65% of the possible morphospecies in the community (Figure 1a). This is within expectations when compared to similar studies (Amancio et al., 2019; Rhoades et al., 2017).

These 390 morphospecies belonged to seven classes, 24 orders, 118 families, and 198 genera of arthropods (Table S2). We were able to amplify *CO1* gene for 314 morphospecies. This was probably due to nucleic acid degradation as they were brought out of storage many times for visual identification, sorting, and photography and also underwent several washes in ethanol. Most of these samples were of single individuals (190 morphospecies) which prevented DNA extraction from additional samples.

Out of 390 morphospecies screened, 47 (12.05%) were found to be infected with *Wolbachia*. Among these, 38.30% of them belonged to Hymenopterans, 25.53% to Hemiptera, 12.77% to Diptera, 8.51% each to Araneae and Coleoptera, 4.26% to Orthoptera, and 2.13% to Sarcoptiformes (Figure 1b). Two morphospecies, morph0081 and morph0085 (both Hymenoptera—Platygastridae), had multiple *Wolbachia* infections and therefore were not included for further analysis. There were nine infected morphospecies for which we were unable to amplify all the five MLST genes probably because of the above‐mentioned DNA quality issues (Table S2). Since we screened only one individual per morphospecies, it is plausible that many infections were not detected as endosymbiont frequencies rarely reach fixation in host population. Therefore, the frequencies of endosymbionts in our study are a conservative estimate of actual infection rates.

We proceeded with the 36 unique host‐*Wolbachia* combinations and 34 unique STs for which we could amplify all the five MLST genes. When resultant 180 allele profiles were compared with the existing sequences in PubMLST database, 77 new allelic profiles (14 each for *gatB* and *coxA*, 27 for *hcpA*, 12 for *ftsZ,* and 10 for *fbpA*) with 30 new STs were found (Table 2). For the strains labeled as ST‐N1 and ST‐N2, unique STs could not be assigned through PubMLST, as only one strand of *gatB* (ST‐N1, ST‐N2) and *ftsZ* (ST‐N1) could be amplified (Table S2). As PubMLST requires chromatogram information from both strands, these were manually labeled as ST‐N1 and ST‐N2.

**TABLE 2:** Allele profiles of MLST genes for 36 unique host‐*Wolbachia* combinations

Sample ID	Class	Order	Family	Genus	*gatB*	*coxA*	*hcpA*	*ftsZ*	*fbpA*	ST	Supergroup
Morph0023	Insecta	Hymenoptera	–	–	near 301	**303**	**317**	near 32	353	**ST‐N1**	A
Morph0076	Arachnida	Araneae	Phrurolithidae	*Orthobula*	**310**	7	**320**	32	**471**	**ST‐544**	A
Morph0080	Insecta	Diptera	Phoridae	*Dohrniphora*	46	288	**321**	**266**	**472**	**ST‐547**	A
Morph0082	Insecta	Hymenoptera	Platygastridae	–	**311**	**304**	**322**	**267**	**473**	**ST‐548**	A
Morph0095	Insecta	Hymenoptera	Formicidae	*Cardiocondyla*	3	**305**	**356**	**258**	**474**	**ST‐550**	A
Morph0152	Insecta	Hymenoptera	Platygastridae	*Dicroscelio* sp.	**313**	241	**323**	3	17	**ST‐553**	A
Morph0171	Insecta	Hymenoptera	Diapriidae	*Trichopria* sp.	87	111	103	70	120	**ST‐554**	A
Morph0182	Insecta	Hymenoptera	Platygastridae	*Idris* sp.	**314**	**307**	**324**	3	120	**ST‐555**	A
Morph0189	Insecta	Hymenoptera	Bethylidae	*Laelius*	22	**308**	24	3	23	**ST‐556**	A
Morph0269	Insecta	Hymenoptera	Platygastridae	*Scelio* sp.	**315**	**311**	**328**	**271**	**477**	**ST‐562**	A
Morph0293	Insecta	Hymenoptera	Platygastridae	*Telenomus*	**316**	**313**	**330**	**272**	351	**ST‐564**	A
Morph0294	Insecta	Hymenoptera	Platygastridae	*–*	**317**	7	**344**	**273**	**479**	**ST‐565**	A
Morph0324	Insecta	Hymenoptera	Platygastridae	–	**322**	**313**	**330**	**272**	351	**ST‐575**	A
Morph0330	Arachnida	Araneae	Uloboridae	*Uloborus*	**319**	**315**	1	3	217	**ST‐567**	A
Morph0352	Insecta	Coleoptera	Corylophidae	–	**320**	15	**348**	6	17	**ST‐569**	A
Morph0375	Insecta	Coleoptera	Chrysomelidae	*Monolepta*	**321**	**316**	**349**	154	122	**ST‐570**	A
Morph0376	Insecta	Hymenoptera	Formicidae	*Pheidole*	3	**305**	**350**	**258**	**474**	**ST‐571**	A
Morph0001	Insecta	Orthoptera	Gryllidae	*Neonemobius*	9	224	30	20	25	**ST‐541**	B
Morph0098	Insecta	Hemiptera	Aphididae	*Phorodon*	9	224	30	20	25	**ST‐541**	B
Morph0213	Insecta	Hemiptera	Psyllidae	*Heteropsylla*	250	66	88	15	417	**ST‐559**	B
Morph0220	Insecta	Hemiptera	Delphacidae	*–*	250	66	88	15	417	**ST‐559**	B
Morph0009	Insecta	Hemiptera	Pyrrhocoridae	*Dysdercus*	**309**	287	**316**	**265**	7	**ST‐542**	B
Morph0026	Insecta	Orthoptera	Gryllidae	*Loxoblemmus*	9	224	**318**	20	25	**ST‐543**	B
Morph0111	Insecta	Hemiptera	Delphacidae	*Nilaparvata*	107	87	29	35	27	ST‐163	B
Morph0210	Insecta	Coleoptera	Chrysomelidae	*Bruchus*	39	**310**	**326**	**270**	27	**ST‐558**	B
Morph0214	Insecta	Hemiptera	Delphacidae	*Muellerianella*	9	2	**327**	36	9	**ST‐560**	B
Morph0285	Insecta	Diptera	Phoridae	–	39	14	40	36	4	ST‐41	B
Morph0288	Insecta	Hemiptera	–	–	9	**312**	**329**	7	**478**	**ST‐563**	B
Morph0329	Insecta	Hemiptera	Cicadellidae	*Balclutha*	**318**	**314**	**345**	**274**	**480**	**ST‐566**	B
Morph0343	Insecta	Hymenoptera	Encyrtidae	–	near 217	7	**346**	**275**	359	**ST‐ N2**	B
Morph0348	Insecta	Coleoptera	Chrysomelidae	–	16	14	**347**	23	4	**ST‐568**	B
Morph0381	Insecta	Hemiptera	Cicadellidae	*Agalliopsis*	109	87	**351**	35	27	**ST‐572**	B
Morph0386	Insecta	Hemiptera	Cicadellidae	–	126	66	**352**	15	136	**ST‐573**	B
Morph0396	Insecta	Hemiptera	Lygaeidae	*Nysius*	16	14	**353**	73	4	**ST‐574**	B
Morph0148	Arachnida	Araneae	Gnaphosidae	*Zelotes*	**312**	**306**	**319**	**268**	**475**	**ST‐552**	F
Morph0206	Insecta	Hymenoptera	Formicidae	*Paratrechina*	73	**309**	**325**	**269**	**476**	**ST‐557**	F

Note

Bold numbers represent new alleles and STs.

**FIGURE 2:**
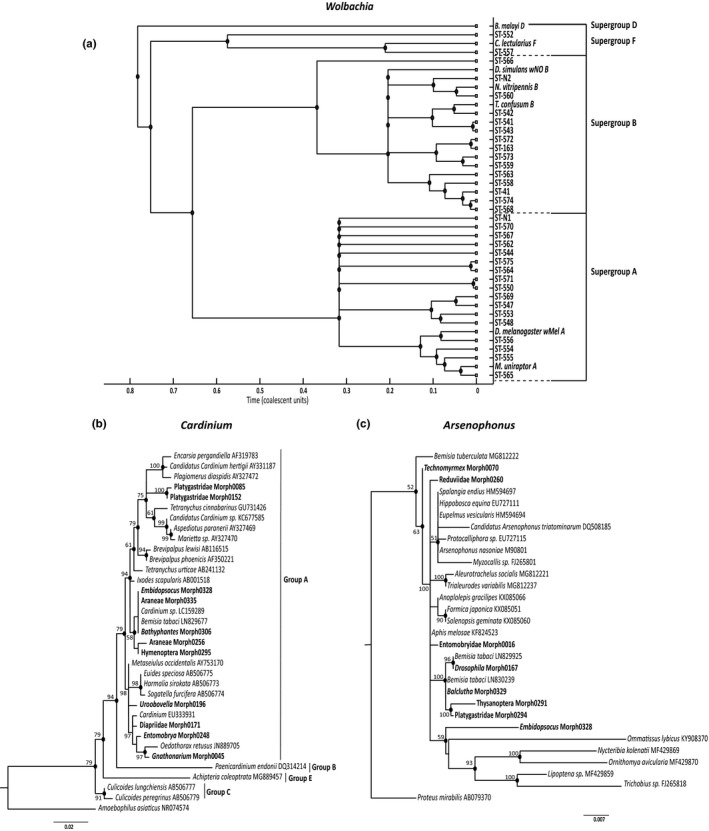
Phylogenetic analysis of (a) *Wolbachia*, (b) *Cardinium,* and (c) *Arsenophonus* found, shown with some known sequences for better resolution. *Wolbachia* phylogenetic tree was constructed using MLST data in ClonalFrame with at least 50% majority rule consensus. *Cardinium* and *Arsenophonus* phylogeny was made in MEGA7 using 16*S rRNA* gene fragment using K2+g substitution model. Numbers on the nodes represent clade credibility values. *Wolbachia* infections are shown as STs whereas *Cardinium* and *Arsenophonus* are labeled with the host taxa that they infected. Infections obtained in this study are in bold. *Brugia malayi, Amoebophilus asiaticus,* and *Proteus mirabilis* were taken as out‐group for *Wolbachia, Cardinium,* and *Arsenophonus* phylogenetic analyses, respectively

Phylogenetic analysis of the MLST data, using ClonalFrame, showed 17 *Wolbachia* strains from supergroup A, 15 from B supergroup and two strains from supergroup *F* (Figure 2a). Supergroup A infections were predominantly found in Hymenoptera (70.5%) whereas Hemipterans had mostly B supergroup infections (73.3%). Such host taxonomic bias of *Wolbachia* supergroups has been noted previously in Hymenopterans such as parasitoid wasps (Mohammed et al., 2017), ants (Russell et al., 2009), and bees (Gerth et al., 2013), in Hemipterans (Bing et al., 2014; Li et al., 2017), lepidopterans (Duplouy & Hornett, 2018; Ilinsky & Kosterin, 2017), and also in Dipterans (Stahlhut et al., 2010).

Eleven (2.82%) of the morphospecies had *Cardinium* infections with four (33%) each from Araneae and Hymenoptera, and one each from Entomobryomorpha, Mesostigmata, and Psocodea (Figure 2b). All the 11 *Cardinium* strains found in this study clustered with group A *Cardinium* (Nakamura et al., 2009). Three morphospecies, morph0085 (Hymenoptera—Platygastridae), morph0152 (Hymenoptera—*Dicroscelio* sp.), and morph0171 (Hymenoptera—*Trichopria* sp.) were found to be infected with both *Wolbachia* and *Cardinium*. Eight morphospecies (2.05%) had *Arsenophonus* infections with two each from Hemiptera and Hymenoptera and one each from Diptera, Entomobryomorpha, Psocodea, and Thysanoptera (Figure 2c). Two morphospecies, morph0294 (Hymenoptera—Platygastridae) and morph0329 (Hemiptera—*Balclutha*), were found to be infected with both *Wolbachia* and *Arsenophonus*. Morph0085 had multiple *Wolbachia* as well as *Cardinium* infections, whereas Morph0328 (Psocodea—*Embidopsocus*) had both *Cardinium* and *Arsenophonus*.

### 3.2 Horizontal transfer of endosymbiont strains

**FIGURE 3:**
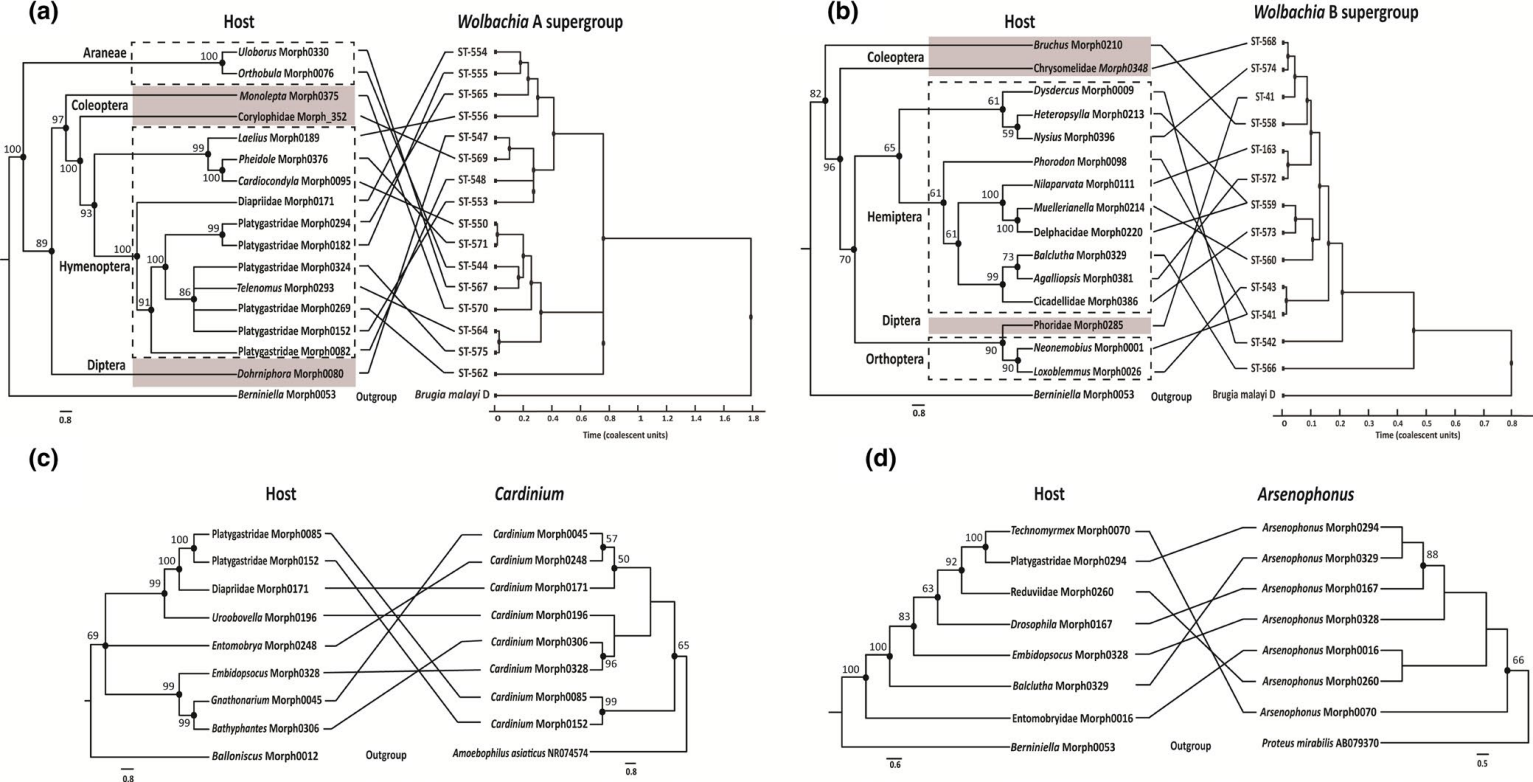
Association between infected host (left) and endosymbiont (right) phylogeny with (a) *Wolbachia* A supergroup, (b) *Wolbachia* B supergroup, (c) *Cardinium,* and (d) *Arsenophonus*. Host phylogeny was constructed in MrBayes using *CO1* fragments. Phylogenetic relationship between different *Wolbachia* strains was interpreted through ClonalFrame with at least 50% majority rule consensus. *Cardinium* and *Arsenophonus* 16*S rDNA* phylogeny was constructed using MEGA7. Black dots in endosymbiont tree represent clade credibility >50

The comparison of the phylogenies of host and their corresponding endosymbionts (Figure 3) suggests horizontal transfer of the endosymbionts within the soil arthropods. This was confirmed by a lack of significant correlations by Mantel test (*r*) (entire *Wolbachia* dataset‐ *r* = 0.205, *p* = 0.002; *Wolbachia* A supergroup‐ *r* = 0.082, *p* = 0.234; B supergroup‐ *r* = 0.233, *p* = 0.089; *Cardinium*‐ *r* = 0.107, *p* = 0.309; *Arsenophonus*‐ *r* = 0.315, *p* = 0.209) between pairwise distances of host and their corresponding endosymbiont (Figure S5). However, PACo analysis showed some cophylogenetic pattern (*p* < 0.05) between host and their corresponding endosymbiont within all datasets (entire *Wolbachia* dataset‐ m^2^xy = 0.288, *p* = 0; *Wolbachia* A supergroup‐ m^2^xy = 0.49, *p* = 0; B supergroup‐ m^2^xy = 0.52, *p* = 0.04; *Cardinium*‐ m^2^xy = 0.19, *p* = 0.01; *Arsenophonus*‐ m^2^xy = 0.17, *p* = 0.04; Figure S6), but ParaFit results suggest the same only for the entire *Wolbachia* dataset (ParaFit Global = 0.083, *p* = 0.002) and *Wolbachia* supergroup A (ParaFit Global = 0.001, *p* = 0.032). Evidence for cospeciation events was rejected (Table S3) for *Wolbachia* supergroup B (ParaFit Global = 0.001, *p* = 0.19), *Cardinium* (ParaFit Global = 9.67E‐05, *p* = 0.05), and *Arsenophonus* (ParaFit Global = 6.97E‐0.05, *p* = 0.09). Furthermore, it was not clear which host–endosymbiont links are contributing to overall congruency because PACo results differ from ParaFitLink1 and ParaFitLink2 (Table S3). The inconsistency between PACo and ParaFit analyses therefore indicates there is no unambiguous signal of codependence of host and endosymbiont phylogeny. However, if endosymbionts are first moving around the host taxa of this particular community, then very similar bacterial strains would be found in taxonomically distant soil arthropods. This is precisely what we found with two distinct *Wolbachia* strains. ST‐541 and ST‐559 were each found in two unrelated hosts (Table 2). Morph0001 (Orthoptera—*Neonemobius*) and morph0098 (Hemiptera—*Phorodon*) were both found to be infected with *Wolbachia* ST‐541, whereas ST‐559 was found in both morph0213 (Hemiptera—*Heteropsylla*) and morph0220 (Hemiptera—Delphacidae). Again, the possibility remains that these transfers could have happened independently and not correlated with the hosts being members of a particular community, but this assumes a nonparsimonious explanation that two independent events would converge on the transfer of the same *Wolbachia* ST in two different hosts.

Thus, horizontal transfer can also create opportunities where endosymbionts can potentially undergo recombination with each other since they are now in the same host cytoplasm.

### 3.3 Recombination events between endosymbiont strains

Recombination in endosymbiont genomes is pervasive and can significantly increase diversification of these bacteria (Jiggins et al., 2001). To check for incidence of recombination, we first analyzed the overall rates of recombination in the *Wolbachia* sequences with both ClonalFrame and RDP4. Both analyses showed a rate of nucleotide substitutions due to recombination/point mutation (*r/m*) of around 2.4 (95% confidence interval between 1.4 and 3.7) which represents intermediate rates of recombination (Vos & Didelot, 2009). This also indicates that recombination introduced twice more nucleotide substitutions, as compared to point mutations, in the *Wolbachia* datasets. Unsurprisingly, the Φ test in SplitsTree also showed significant evidence of recombination (*p* < 0.001) for the same *Wolbachia* sequences (Figure S3). However, for *Cardinium* and *Arsenophonus*, RDP4 did not indicate any evidence of recombination. This was probably due to the use of a single gene (16*S rRNA* gene) for these two bacteria.

**FIGURE 4:**
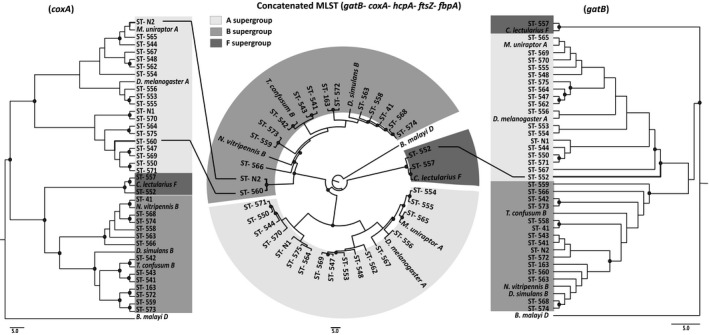
Maximum‐likelihood phylogenetic trees of *coxA* (left), concatenated MLST dataset (center), and *gatB* (right) gene made in MEGA7 using HKY+g, T92+g+I, T92+g substitution models, respectively. Black dots represent bootstrap value >50. *Wolbachia* ST‐N2 and ST‐560 clustered with B supergroup in concatenated MLST phylogenetic tree whereas these strains clustered with A supergroup in the *coxA* phylogenetic tree, indicating recombination between *Wolbachia* supergroups A and B. Similarly, ST‐552 clustered with F supergroup in concatenated MLST tree, but the individual *gatB* gene tree shows it to be from the A supergroup, indicating recombination between A and F supergroups. These three cases (ST‐N2, ST‐560, and ST‐552) represent between‐supergroup recombination of gene or gene segment

To enumerate the recombination events within the *Wolbachia* sequences, we first looked at the phylogenetic trees to check whether single‐gene phylogenies of all the five MLST genes (Figure S4) differ significantly with the concatenated MLST trees (Figure 4). Next, sliding‐window algorithms in RDP4 were used to locate recombination breakpoints. The recombination events detected were then evaluated and confirmed manually. These analyses yielded several putative recombination events elaborated below.

#### 3.3.1 Recombination between supergroups

Three cases of acquisition of a gene or gene segments from different supergroups were detected. Phylogenetic and network analysis of concatenated MLST dataset (Figure 4) showed *Wolbachia* ST‐N2, infecting morph0343 (Hymenoptera—Encyrtidae), to cluster with B supergroup, but individual gene trees revealed that the *coxA* fragment of ST‐N2 clusters with A supergroup (Figure 4) and has the allelic profile of 7. This phylogenetic disparity suggests that *coxA* gene of ST‐N2 was acquired via recombination from a supergroup A *Wolbachia*. Curiously enough, *coxA* allele 7 is also found in two other *Wolbachia*‐infected hosts, ST‐565 of morph0294 (Hymenoptera—Platygastridae) and ST‐544 of morph0076 (Araneae—*Orthobula*), both with supergroup A infections (Table 2). Although it is impossible to know which *Wolbachia* strains originally underwent recombination and gave rise to the recombinant allele 7 of *coxA*, yet the presence of the same allele within the community suggests that the recombination event could have involved members within this ecological community.

Similarly, another case of recombination was observed where a B supergroup *Wolbachia* ST‐560, of morph0214 (Hemiptera—*Muellerianella*), had the *coxA* gene fragment (allele profile 2) from the A supergroup (Figure 4). This recombinant *coxA* allele 2 also shares sequence similarity with ST‐550 and ST‐571, where the *coxA* alleles are different by only two base pairs (*coxA* allele profile 305) indicating that perhaps this is also another case of recombination happening within the community.

Another case of recombination between supergroups was found with another MLST gene, *gatB*, but between supergroups A and F. The *Wolbachia* ST‐552 (supergroup F), infecting morph0148 (Araneae—*Zelotes*), had a recombinant *gatB*, where the last 190 bp fragment came from the A supergroup. As the concatenated MLST tree (Figure 4) shows, ST‐552 clusters with F supergroup, but the individual *gatB* gene tree shows it to be from the A supergroup. This 190 bp fragment differs by only one base pair with ST‐544 infecting morph0076 (Araneae—*Orthobula*). This is also indicative of a possible recombination between these two *Wolbachia* STs belonging to two different supergroups.

#### 3.3.2 Recombination within supergroups

The pervasive recombination necessitated the development of the MLST scheme for *Wolbachia* (Baldo et al., 2006) as single‐gene phylogenies were unable to properly represent the evolutionary history of a particular strain. In this scheme, alleles of any of the five different genes are given the same nomenclature if they share sequence identity. As Table 2 shows, many of the morphospecies also share the same alleles. In fact, instead of the maximum possible number of unique alleles (180) that could have been present across the five MLST loci of the 36 infected morphospecies, there is only 136. This is indicative of acquisition of the same alleles by recombination and is therefore examples of within‐supergroup recombination events whereby MLST fragments are exchanged across endosymbionts.

Next, we tried to identify intergenic (*i.e*., within a particular MLST gene) recombination happening within a supergroup. This detection was done through the different algorithms present in RDP4, most of which uses inherently conservative sliding‐window scans checking for above than expected sequence divergence in the alignments (Martin et al., 2015). Therefore, recombination events happening between closely related strains and/or between regions with low variation will not be recorded as significant events. However, there can be two types of intergenic recombination events. First, different MLST fragments (*e.g*., between *coxA* and *gatB* of two different strains) can combine to form a chimeric gene, and secondly, recombination can happen within the same MLST genes (*e.g*., within *coxA* of two different strains). Our analysis did not find any examples of the former. This is unsurprising as all the MLST fragments are housekeeping genes, and such chimeric variants will be under strong negative selection. However, eight instances of recombination within the same MLST gene were found (Table 3), all within supergroup A.

**TABLE 3:** Recombination events detected in the *Wolbachia* MLST sequences. Putative breakpoints indicate concatenated sequences of MLST genes in the order: *gatB‐coxA‐hcpA‐ftsZ‐fbpA*. *p*‐value was kept at <.01

Recombination event		STs potentially involved	Gene: Putative breakpoints	Detection methods positive for recombination
Between supergroup	A and B supergroups	ST‐N2/565	*coxA*: 370‐771	*RDP, GENECONV, BootScan, MaxChi, Chimera, SiScan*
	A and B supergroups	ST‐560/550	*coxA:* 370‐771	*RDP, GENECONV, BootScan, MaxChi, Chimera*
	A and F supergroups	ST‐544/552	*gatB:* 179‐369	*RDP, GENECONV, BootScan, 3Seq*
Within supergroup	A supergroup	ST‐565/555	*gatB:* 255‐369	*MaxChi, SiScan, 3Seq*
			*fbpA:* 1650‐1800	
	A supergroup	ST‐547/564	*gatB:* 118‐369	*MaxChi, Chimera, SiScan, 3Seq*
			*hcpA:* 771‐885	
	A supergroup	ST‐570/550	*hcpA:* 998‐1215	*MaxChi, Chimera, SiScan*
			*fbpA:* 1650‐1988	
	A supergroup	ST‐544/570	*fbpA:* 1650‐1988	*MaxChi, SiScan, 3Seq*
	A supergroup	ST‐N1/567	*gatB:* 80‐369	*Chimera, SiScan, 3Seq*

#### 3.3.3 How similar are endosymbiont strains within the community?

If an ecological community is the primary site of horizontal transfer of endosymbionts, then the same (or very similar) bacterial strains would be found in multiple host taxa. This essentially means that a few endosymbiont strains within that community will show high incidence than other strains. This would result in a lower estimate of pairwise divergence among the endosymbionts. Using the model from Baldo et al. (2008), we tested whether there is a significant reduction in the expected pairwise divergence of the endosymbionts infecting the soil arthropod community. From our sampling, we found 17 unique STs for *Wolbachia* supergroup A and 15 for *Wolbachia* supergroup B infections, whereas from the PubMLST database, we obtained 228 unique STs for A supergroup and 252 STs for B supergroup (last checked May 2019). Similarly, for the nine samples infected with *Cardinium* and eight for *Arsenophonus*, 248 and 228 sequences, respectively, were obtained from NCBI (last checked May, 2020). Results indicate that mean pairwise distance of *Wolbachia* supergroup A within the community (2.67%) was significantly lower (Wilcox rank‐sum test, *p* < 0.05) than expected (mean = 3.54%; Table S4) and also significantly lower from the mean of all the supergroup A strains present in the PubMLST database (3.69%; Figure S7). In contrast, the mean pairwise distance of *Wolbachia* supergroup B strains within the community (4.17%) was significantly higher (Wilcox rank‐sum test, *p* < 0.05) from both the expected (mean = 3.38%) and the mean of all the B supergroup strains in the PubMLST database (3.43%). These higher than expected values for *Wolbachia* B supergroup strains can indicate the presence of more divergent strains as compared to *Wolbachia* A supergroup within this community. However, when all the *Wolbachia* supergroup infections were taken together and their mean pairwise distance (8.68%) was compared with all such strains in the PubMLST database (8.66%), no significant differences were found (Wilcox rank‐sum test, *p* > 0.05). This perhaps indicates that although the soil arthropod community yielded several unique *Wolbachia* infections (Table 2), on average, this still represents a subset of the *Wolbachia* diversity reported till now. Similar to *Wolbachia* supergroup A, *Cardinium* strains also showed a similar trend where community mean pairwise distance (1.41%) was significantly less (Wilcox rank‐sum test, *p* < 0.05) than expected (mean = 2.48%) and mean of strains obtained from the database (2.01%). However, mean pairwise distance of *Arsenophonus* strains within the community (1.19%) was not significantly different (*p* > 0.05) from the expected (mean = 1.38%) as well as from mean of strains obtained from the database (1.55%). Thus, *Wolbachia* supergroup A and *Cardinium* strains within the community are more closely related among themselves (Table S4) but not *Wolbachia* supergroup B and *Arsenophonus*.

## 4 DISCUSSION

In this study, we evaluated sequence divergence and incidence of recombination in three major endosymbionts (*Wolbachia*, *Cardinium,* and *Arsenophonus*) to answer whether the ecological community represents the primary seat of their horizontal transfer and diversification. We used soil arthropod community because it is relatively insular and has a relatively high habitat endemicity of the resident hosts. Our main goal was to assess whether community members facilitate the spread of endosymbionts as they themselves come in contact with each other for various ecological interactions. To do so, we compared multigene phylogenies of such endosymbiont surveys from different communities. However, in spite of our extensive literature surveys we could not find any such previous reports. Most surveys of arthropod communities concentrated on the hosts rather than on their endosymbionts (Gonçalves et al., 2012). Some studies such as Kittayapong et al. (2003) and Sintupachee et al. (2006) did uncover the resident endosymbionts but mostly with single genes. This precluded a cogent comparison of endosymbiont diversity and incidence of recombination with the present study. Another set of studies did indeed sample endosymbionts with multigene sequences but concentrated on a few, and not all, host taxa within a community (Bing et al., 2014). Again, such studies are not ideal comparisons with the present one as these were biased toward a few host taxa. To partially overcome this problem, we used statistical models with extensive resampling. We observed that the supergroup A *Wolbachia* infections and *Cardinium* do indeed show less pairwise divergence, than expected, in accordance with our predictions. However, supergroup B *Wolbachia* and *Arsenophonus* infections did not show this pattern. In fact, the former shows more variation than expected whereas *Arsenophonus* shows no significant difference. This indicates that these endosymbionts have different propensity and/or rates of horizontal transfer within the community than supergroup A *Wolbachia* and *Cardinium*. This disparity among the endosymbionts was still detectable in spite of our conservative estimate of the number endosymbionts within the community as we only analyzed hosts with single *Wolbachia* infections. This was primarily done to avoid the difficulty in assigning specific MLST alleles to individual bacterial strains.

### 4.1 Are some endosymbionts more prone to horizontal transfer and recombination?

One explanation for the observed patterns could be the relative ease with which supergroup A *Wolbachia* and *Cardinium* can undergo horizontal transfer and recombination compared with supergroup B *Wolbachia* and *Arsenophonus*. This essentially means that the former two endosymbionts would encounter previously existing bacterial infections within their hosts which would increase opportunities for recombination among the pre‐existing and the new bacterial strains. Recombination would then create newer allele variants. This is indeed borne out by the results in Table 2 which depicts the number of unique alleles found in this study among the *Wolbachia* infections. In all, about 84% (71 out of a possible 85 alleles) of the A supergroup infection are unique, whereas about 75% (56 out of a possible 75 alleles) are unique in B supergroup *Wolbachia* infections. Furthermore, as indicated in Table 3, the number of within‐supergroup recombination detected in the A supergroup strains (8 instances) far outnumbers the B supergroup *Wolbachia*, where none were detected. This is in spite of horizontal transfer of the entire B supergroup STs (ST‐541 and ST‐559) to taxonomically unrelated hosts (Table 2). An expected outcome of such pervasive horizontal transfer and resulting recombination would have been an increase in sequence diversity in the A supergroup strains, especially, if the source of recombination had been infections outside the community. This does not seem to be the case as the A supergroup infection shows less than expected pairwise distance (2.67%) when compared with the B supergroup infections (4.17%). This indicates that the sources of recombination must be from infections within this community. In other words, the standing sequence variation of the A supergroup infections is being partitioned across the community‐wide arthropod taxa into newer recombinants with resulting increase in allele diversity but not overall sequence divergence. Moreover, what follows from this relatively low pairwise divergence of the A supergroup infections is that this horizontal transfer and recombination must have been relatively recent or rapid enough for any post‐recombination sequence variation to accumulate. This indicates that the A supergroup infections are either better at horizontal transfer across the community or are presently undergoing such rapid transfers as has been suggested by Werren et al. (1995). Whether this transfer is due to the presence of dominant strains, which are better at horizontal transfer (Turelli et al., 2018), remains to be investigated with more sequence information from these endosymbionts. The B supergroup *Wolbachia* infections show relatively diverged strains with low rates of recombination indicating more stable infections. Since little is known about the biological characteristics of *Wolbachia* supergroups, other than sequence divergence, it is difficult to speculate whether there are supergroup‐specific effects on their hosts. For example, it is not known whether supergroup infections have different success rates in establishing stable infections after horizontal transfer or whether some are more prone to horizontal transfer? Therefore, we concentrated on specific trophic interactions of the hosts themselves and tried to explain why supergroup A infections show such extensive horizontal spread.

### 4.2 Are parasitoids serving as the conduit for the spread of endosymbionts?

Parasitoids can serve as a driving force for the horizontal transfer of endosymbionts (Haine et al., 2005) as their lifestyle entails close cellular and tissue contact with their host. Horizontal transfer involving parasitoids is generally unidirectional (from host to parasitoid) because they usually end up killing the host. But parasitoids can also act as phoretic vectors and can transmit endosymbionts by sequential probing of infected and uninfected hosts (Ahmed et al., 2015; Gehrer & Vorburger, 2012). Moreover, horizontal transfers can also happen between parasitoids if infected and uninfected parasitoids share the same host (Huigens et al., 2004). Such habits can also facilitate multiple infections if parasitoids infecting same host have different endosymbiont infections. As these multiple infections come in close contact, they can undergo recombination. Such parasitoid‐mediated horizontal transfer could be an explanation for the distribution of A supergroup *Wolbachia* infections in our sampling. Out of the 17 *Wolbachia* A supergroup STs found, nine STs were found in parasitoid wasps (Hymenoptera). Seven STs were found from Platygastridae and one each from Bethylidae and Diapriidae (Table 2). Therefore, the comparatively higher incidence of recombination in *Wolbachia* supergroup A infections could be due to their presence in parasitoid hosts. Similarly, predators and parasites can also be conduits for the spread of endosymbionts as observed during predation of infected *Armadillidium vulgare* by *Porcellio dilatatus* (Le Clec’h et al., 2013). Among the soil arthropod community, we also observed incidences of *Wolbachia* recombination among predators. *Wolbachia* F supergroup ST‐552, infecting morph0148 (Araneae—*Zelotes*), had a fragment of *gatB* gene similar to the A supergroup from ST‐544 which infected morph0076 (Araneae—*Orthobula*). Furthermore, the *fbpA* gene of ST‐544 was found to have probably recombined with ST‐570 (Morph0375, Coleoptera—*Monolepta* sp.). Compared to predation, host–parasitoid interactions generally last longer can take place at various developmental stages and allow endosymbiotic bacteria to enter host through various tissues (Sanaei et al., 2021). These features can increase the probability of parasitoid‐mediated horizontal transfer which remains one of the most common routes for endosymbiont spread (Hou et al., 2020; Morrow et al., 2014). Therefore, occurrence of closely related A supergroup *Wolbachia* among the parasitoid hosts of this community is not unexpected. However, this pattern can also be explained by *Wolbachia* A supergroup infections being “better” at horizontal transfer and subsequent establishment in a new host. Further studies are required to answer which of these two explanations are more plausible.

### 4.3 How are endosymbionts spreading from one ecological community to another?

Ecological communities are a diverse assemblage of many different species involved in a web of interactions with each other (Agrawal et al., 2007). However, rarely, such communities remain isolated from each other. There are certain members which are relatively cosmopolitan and interconnect with members of multiple communities (Stireman & Singer, 2003) leading to a metacommunity‐wide distribution (Brown et al., 2020). This cosmopolitan distribution of these arthropods can lead to horizontal transfer of their resident endosymbionts to different ecological communities. Within the soil arthropod community, we have found one such example which can potentially be a source of horizontal transfer of endosymbionts across many other communities. The macropterous form of the planthopper *Nilaparvata lugens* (morph0111, BOLD ID SAEVG089‐20; Table S2) was found from the leaf litter. *N. lugens* is a highly destructive pest of rice across tropical Asia and can also survive on other tropical grass species (Khan et al., 1988). It is known to migrate long distances in search of actively growing rice plants (Riley et al., 2003). The presence of *N. lugens* is unsurprising as our sampling season (October) coincided with the rice harvesting season in Northwest India. *N. lugens* is known to be infected with several endosymbionts such as *Wolbachia* and *Arsenophnonus* (Qu et al., 2013). In this study, it was found to be infected with *Wolbachia* ST‐163 from the B supergroup. The same *Wolbachia* ST has also been reported in *N. lugens* from Southern China (Zhang et al., 2013). This indicates that such invasive pest species can potentially introduce their resident endosymbionts into many different arthropod communities.

Conversely, the presence of very similar endosymbionts in geographically distinct locations can indicate their spread from one ecological community to another. The *Wolbachia* B supergroup, ST‐41, was found to infect a phorid fly (morph0285). The same ST‐41 has been found in calyptrate flies (Stahlhut et al., 2010) as well as from several other lepidopterans (Ilinsky & Kosterin, 2017; Narita et al., 2011; Russell et al., 2009; Salunke et al., 2012). This is not unexpected given the diversity of *Wolbachia* infections. However, what is unexpected is the location of the hosts with ST‐41 ranges from North America, Africa, Russia, South Asia, and South‐Eastern Asia all the way to Japan. Unfortunately, it is difficult to conjecture about the reasons behind such a huge range, as corroborating community‐wide data are lacking.

The above two instances testify to the utility of a MLST‐based approach to understand *Wolbachia* diversity and spread across global arthropod communities (Wang et al., 2020). Moreover, these cases also highlight the importance of collecting community‐wide data to understand the probable chain of transfer of these bacteria. Such data can also illuminate similar connections for the spread of *Arsenophonus* and *Cardinium* if employed with multilocus data (Jousselin et al., 2013; Stouthamer et al., 2019).

A major goal of endosymbiont research is to explain the tempo and mode of their spread across arthropod communities across the world. We contend that evaluating endosymbiont diversity within specific ecological communities is the key to understand this spread. Such studies would give us specific examples of bacterial strains that are better at spreading as well as uncover specific ecological roles of arthropod hosts which are more amenable to horizontal transfer of their resident endosymbionts. As data from such studies accumulate specific patterns will emerge which can then be empirically tested.

## CONFLICT OF INTEREST

None declared.

## AUTHOR CONTRIBUTIONS

**Manisha Gupta:** Conceptualization (equal); data curation (lead); formal analysis (lead); investigation (lead); methodology (lead); software (lead); validation (lead); visualization (equal); writing–original draft (equal); writing–review and editing (equal). **Rajbir Kaur:** Investigation (equal); methodology (equal); visualization (equal). **Ankita Gupta:** Data curation (supporting); investigation (supporting). **Rhitoban Raychoudhury:** Conceptualization (lead); formal analysis (equal); funding acquisition (lead); investigation (lead); methodology (lead); project administration (lead); supervision (lead); validation (equal); visualization (equal); writing–original draft (equal); writing–review and editing (equal).

## Supporting information

Supplementary Material

## Data Availability

All sequences obtained from this study were deposited in NCBI. Accession numbers are as follows: for *CO1* data‐MN447522‐MN447531, MN520845‐MN521147, MN901899; *Wolbachia gatB*‐MN594583‐MN594618; *coxA*‐MN594619‐MN594654; *hcpA*‐MN594655‐MN594690; *ftsZ*‐MN594691‐MN594726; *fbpA*‐MN594727‐MN594762; *Cardinium* 16*S*‐MN594564‐MN594574; and *Arsenophonus* 16*S*‐MN594575‐MN594582 (Table S2). *Wolbachia* MLST data were also deposited on PubMLST database having ID ST‐541‐544, 547‐548, 550, 552‐560, 562‐575 (Table 2). Morphospecies images along with their corresponding *CO1* gene sequences were also deposited in BOLD database having process ID SAEVG001‐20: SAEVG314‐20.
