# Supplementary material for: Are ecological communities the seat of endosymbiont horizontal transfer and diversification? A case study with soil arthropod community
Source: Ecol Evol. 2021 Oct 16;11(21):14490–508. doi: 10.1002/ece3.8108 (PMC8571607; doi:10.1002/ece3.8108)
Supplement: Supplementary file 1 — Supplementary Material [file ECE3-11-14490-s001.pdf]

Supporting Information for online Publication

Are ecological communities the seat of endosymbiont horizontal transfer and diversification? A case study with soil arthropod community.

Manisha Gupta[1], Rajbir Kaur[1, 2], Ankita Gupta[3], Rhitoban Raychoudhury[1]*

1: Indian Institute of Science Education and Research, Mohali (IISER-Mohali), Knowledge city, Sector 81, SAS Nagar, Manauli, PO 140306, Punjab, India.

2: Indian Institute of Science, CV Raman Rd, Bengaluru, Karnataka 560012, India.

3: ICAR- National Bureau of Agricultural Insect Resources (NBAIR), Bellary Road, H.A. Farm Post, Hebbal, Bengaluru, Karnataka 560024, India.

*Corresponding author: rhitoban@iisermohali.ac.in

Supplementary figure S1

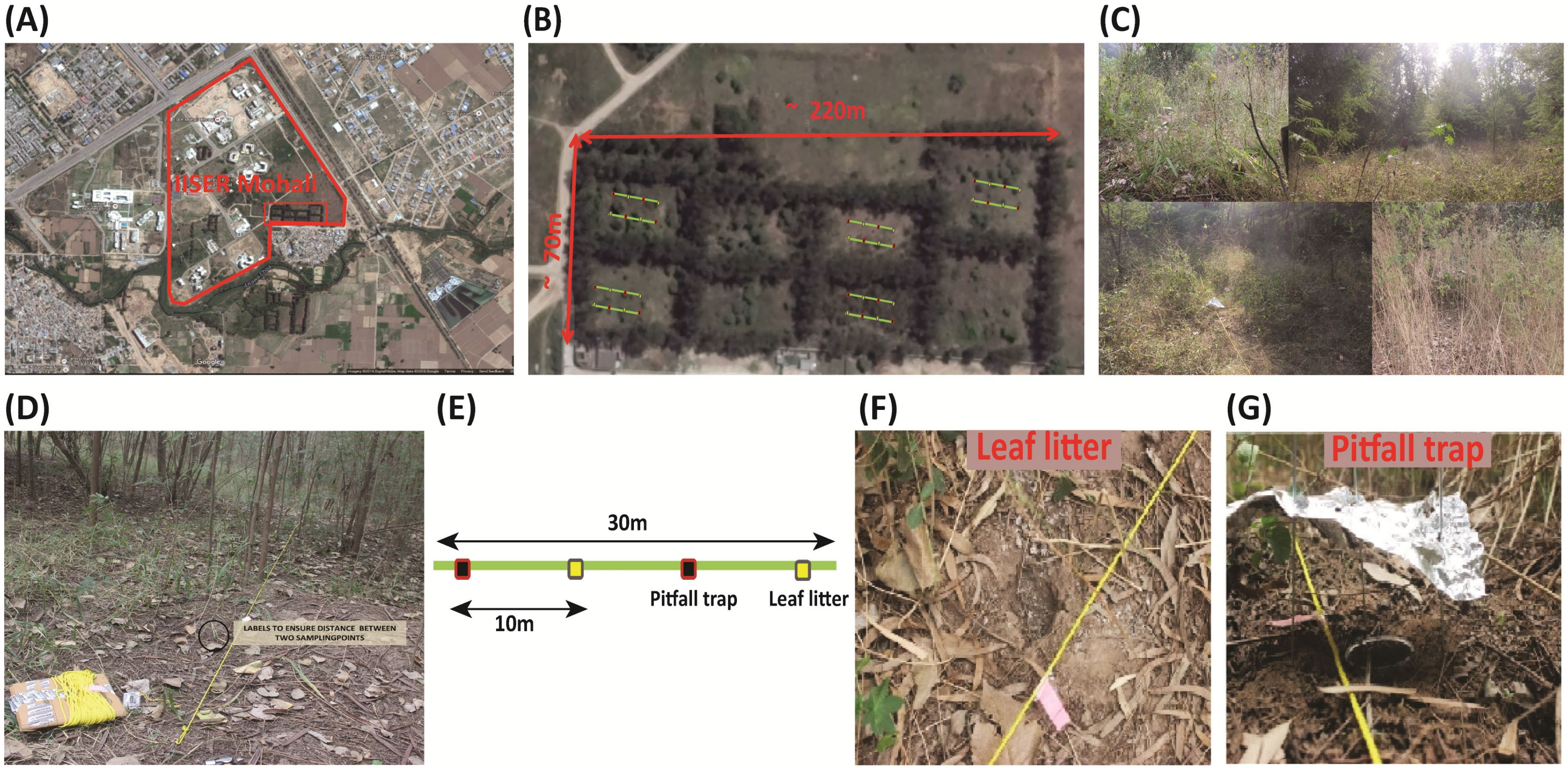

Figure S1: Sampling Plot: The study site had poplar plantations along the perimeter. Sampling was done inside this site. Habitat structure in sampling area was close grassland with some tree plantation. Soil type in sampling region is mostly alluvial, rich in nutrients and suitable for crops, as is the characteristic soil type of this region (http://dcmsme.gov.in/old/dips/SAS%20Nagar.pdf). Sampling site and collection methods: (A) Google map location of sampling site; (B) Sampling plot showing five randomly selected quadrants having two parallel transects in green; (C) Sampling area; D) Sampling region; E) 30m long sampling transect having alternate pitfall traps as red dots and leaf litter sample collection sites in yellow; (F) Leaf litter collection site; (G) Pitfall trap.

Supplementary figure S2

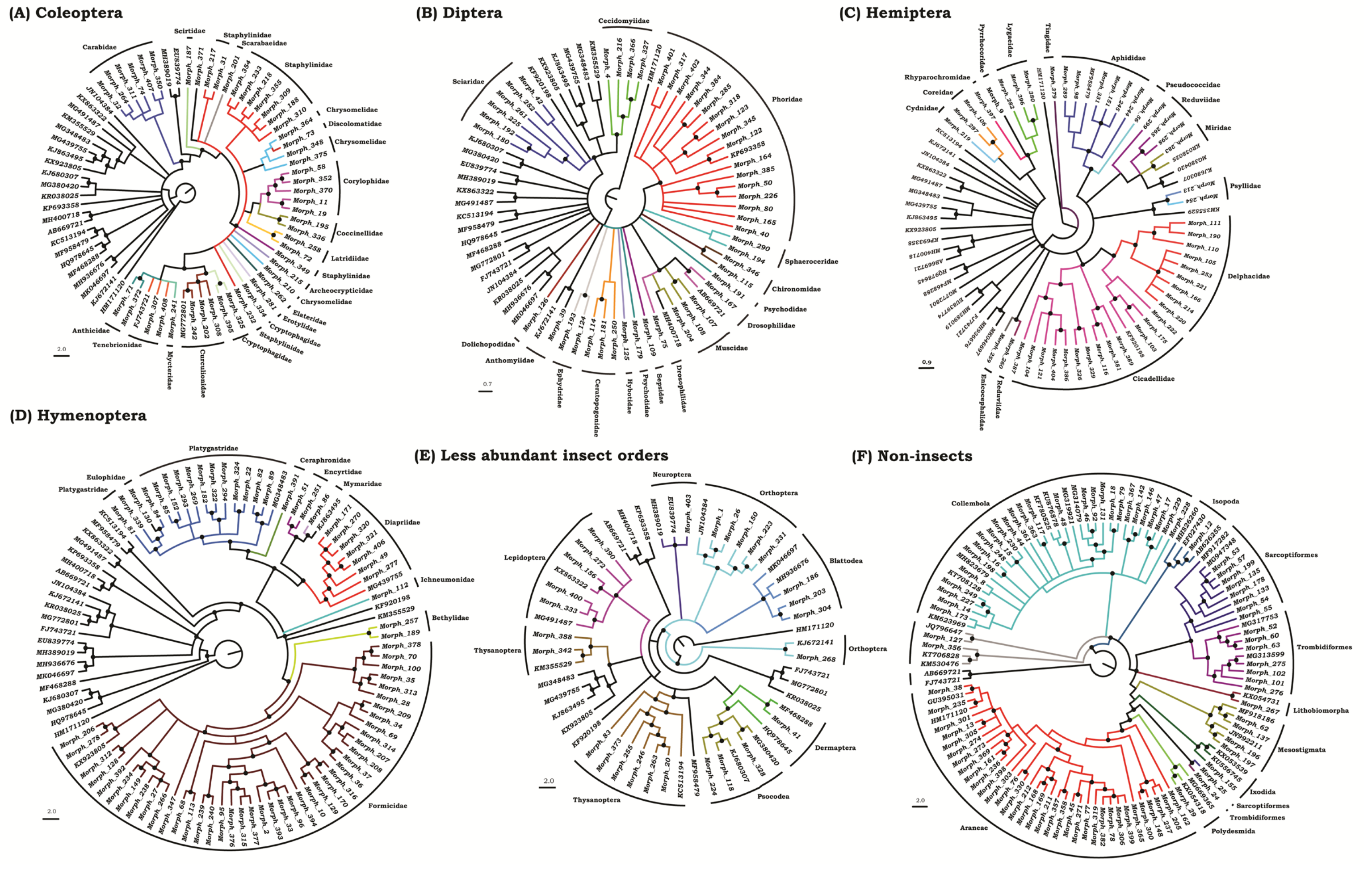

Figure S2: Phylogenetic analysis of different taxa using mitochondrial *CO1* gene sequences shown along with some known sequences from NCBI. Bayesian phylogenetic trees were made in MrBayes v3.2.5 (Ronquist et al., 2012). Black dots represent clade credibility >70. (A) Coleoptera, (B) Diptera, (C) Hemiptera, (D) Hymenoptera, (E) less abundant insect orders (F) Non-insects.

Supplementary figure S3

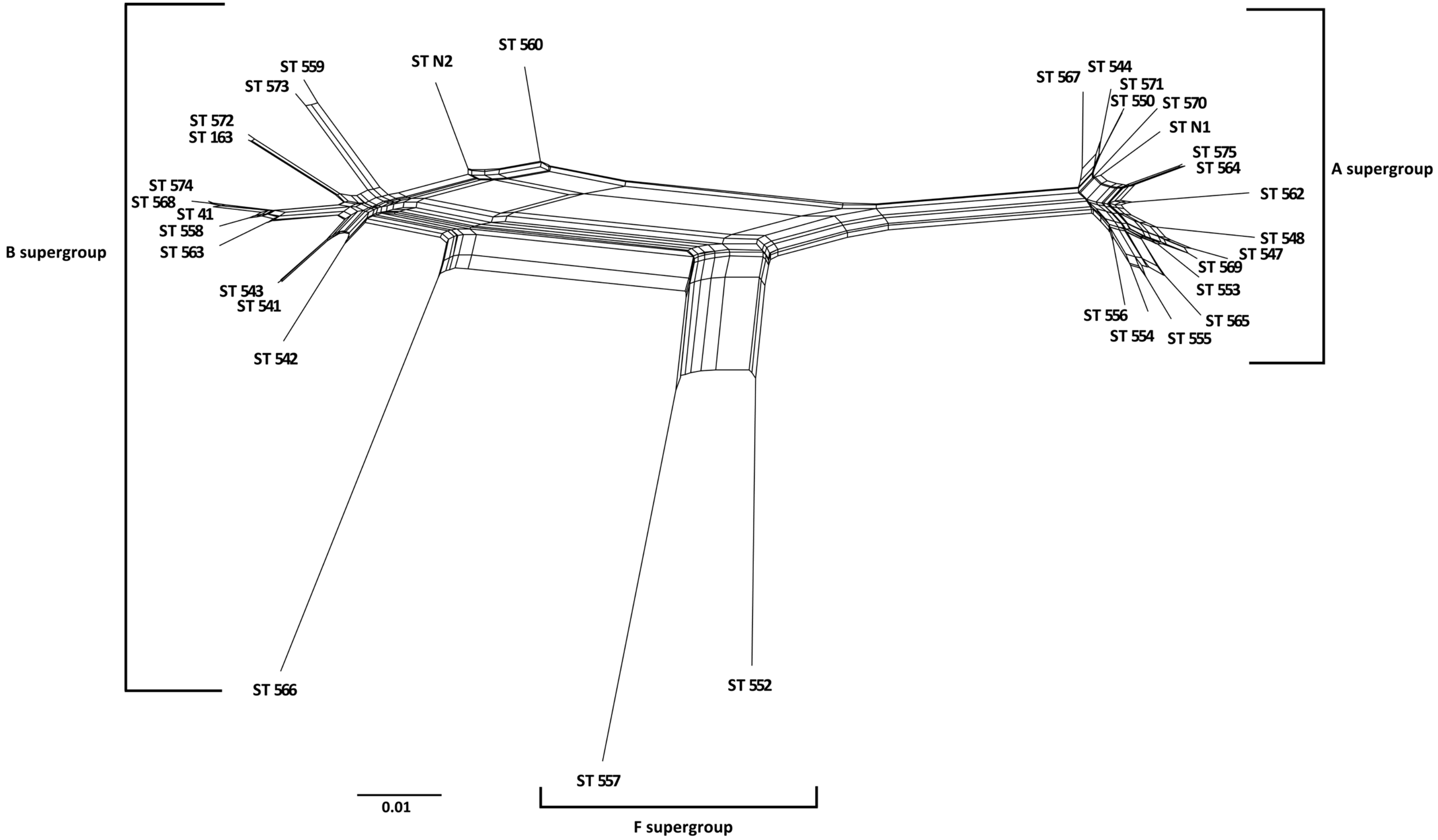

Figure S3: Phylogenetic network of *Wolbachia* concatenated MLST data (2079bp) comprising of *gatB*, *coxA*, *hcpA*, *ftsZ* and *fbpA* gene made through NeighborNet method using SplitsTree v4.14.8 (Huson, Kloepper, & Bryant, 2008).

Supplementary figure S4

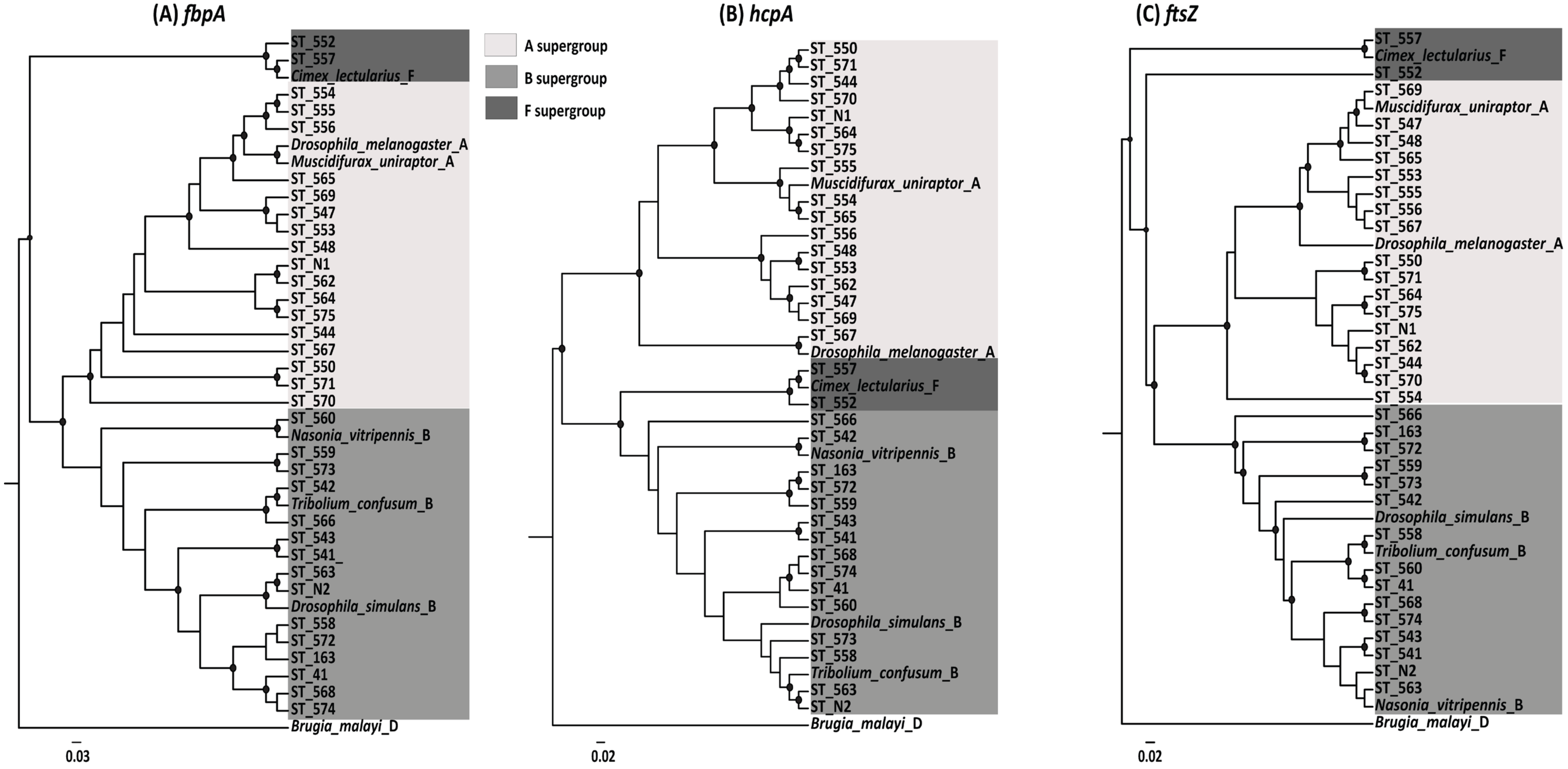

Figure S4: Maximum likelihood phylogenetic trees of *Wolbachia fbpA, hcpA* and *ftsZ* gene made using evolutionary model T92+g in MEGA7. Black dots represent bootstrap value >50.

Supplementary figure S5

A) *Wolbachia*

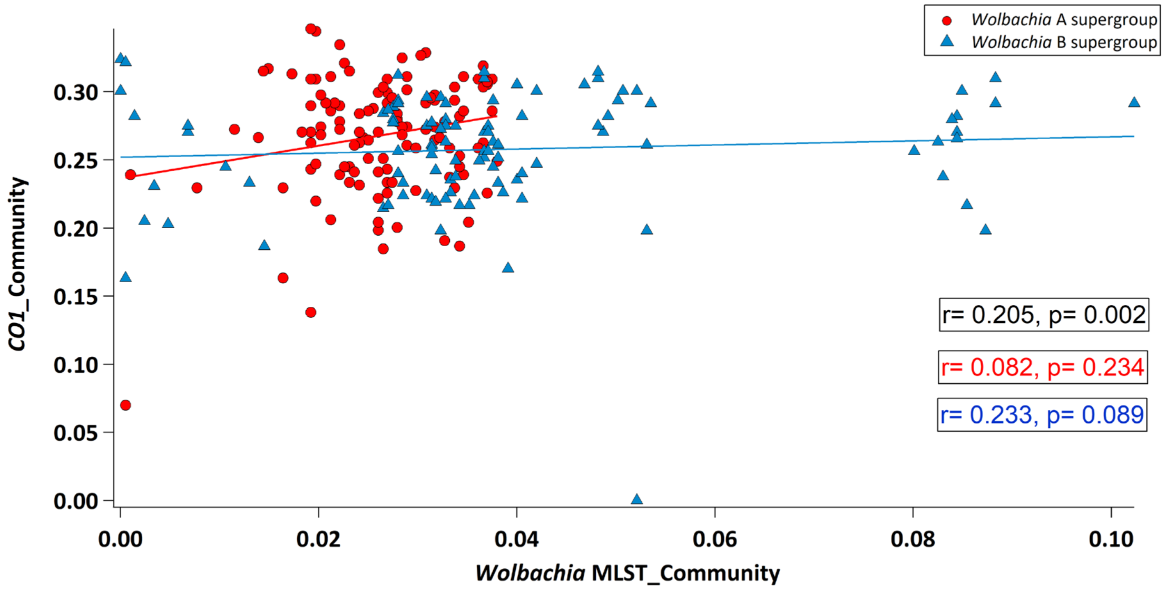

B) *Cardinium*

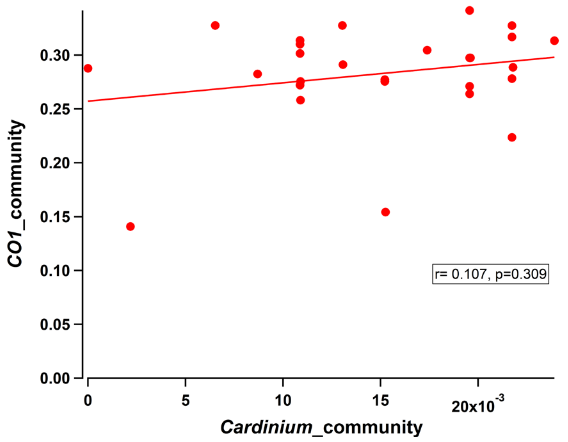

C) *Arsenophonus*

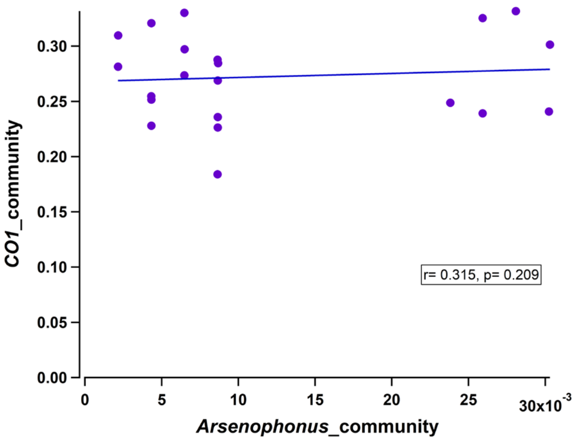

Figure S5: Correlation between pairwise divergence of host (y-axis) and their corresponding endosymbiont (x-axis) is shown. For all datasets, Spearman method of Mantel test (r) shows that there is no correlation between host and its endosymbiont. Analysis for five endosymbiont and host comparison were computed (A) *Wolbachia* supergroup A and their host (red), supergroup B and their host (blue) and all *Wolbachia* taken together with their host (black), (B) *Cardinium* and their host, (C) *Arsenophonus* and their host.

Supplementary figure S6

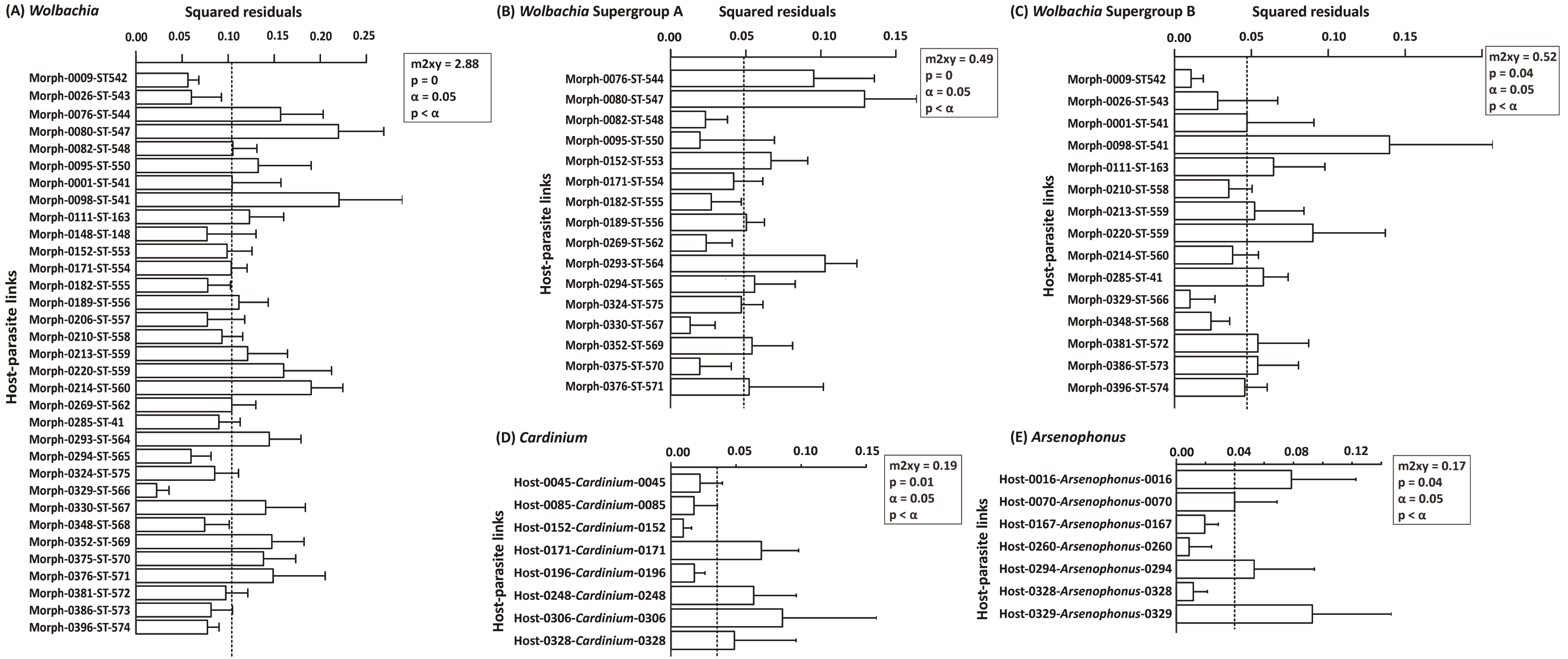

Figure S6: PACO Analysis: Contribution of individual host-symbiont association towards topological congruence between host and endosymbiont. Jackknife squared residual (bars) with 95% confidence intervals (error bars) was calculated for each association. Dashed line indicates the median squared residual value. Host-symbiont association bars above the median line are the ones which are contributing less towards congruency.

Supplementary figure S7

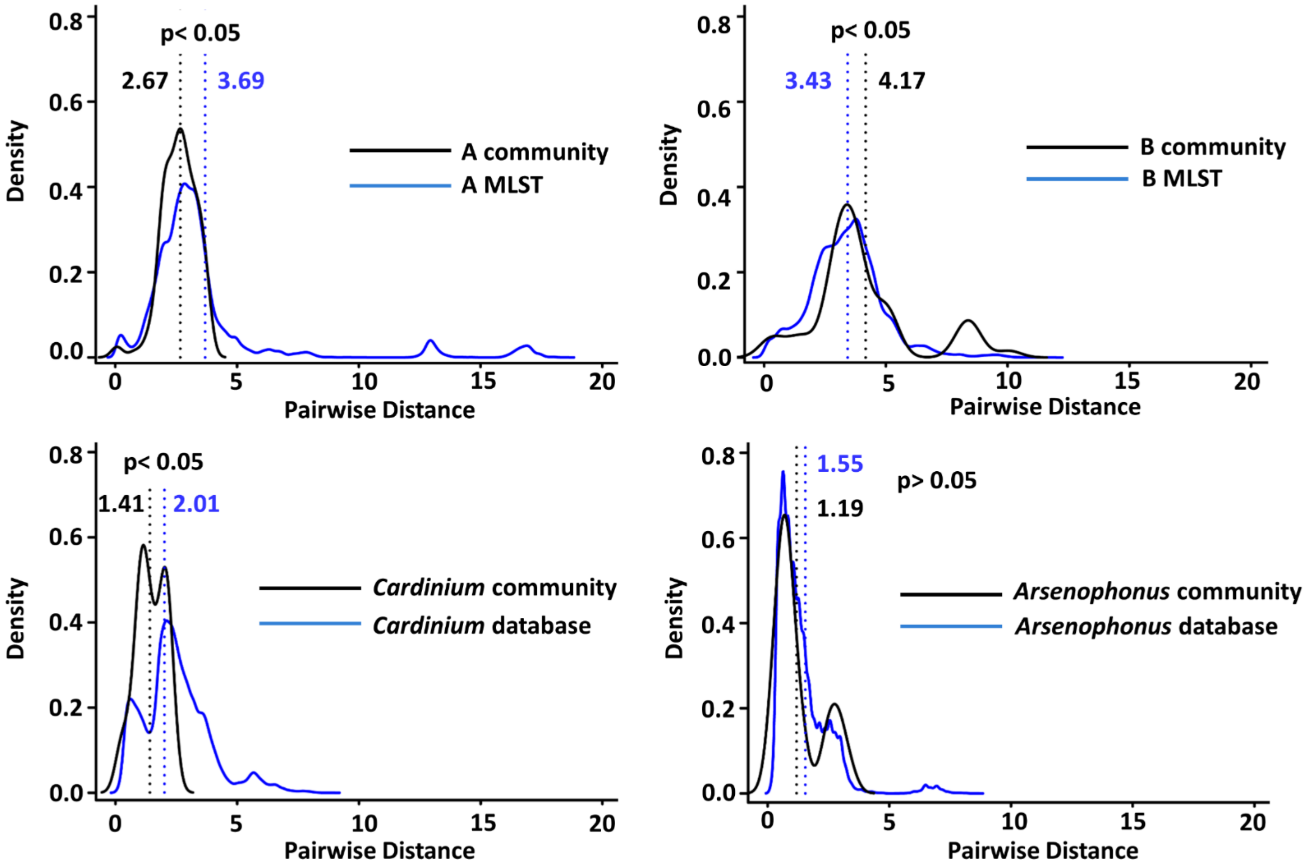

Figure S7: Density plots for endosymbiont divergence (*Wolbachia* A supergroup, B supergroup, *Cardinium* and *Arsenophonus*) from the soil arthropods and their respective databases plotted in R.

Table S1: Species richness of the community represented by different diversity indices computed by EstimateS (Colwell, 2013) reported with their standard deviation.

Mean of different diversity indices	Value
Incidence Coverage Estimator (ICE)	858 (± 0)
Chao 2	743 (± 65.35)
Jack 2	738 (± 0)
Abundance Coverage Estimator (ACE)	701 (± 0)
Chao 1	651 (± 51.97)
Jack 1	600 (± 32.97)

Table S2: Identification of morphospecies using *CO1* gene fragments. Information about their abundence, infection status, data availability and collection site is also given. '*' labelled morphospecies represents infected hosts for which we were unable to amplify the *CO1* fragment. # Represent number of individuals obtained for each morphospecies during sampling. A= *Arsenophonus* infected, C= *Cardinium* infected, W= *Wolbachia* infected, W*= *Wolbachia* positive samples for which we could not amplify all the five MLST genes, U= Uninfected. GenBank accession numbers of *Wolbachia* gene sequences are mentioned in the following order: *gatB; coxA; hcpA; ftsZ; fbpA.*

Morph ID	#	Infection status	Endosymbiont's Accession numbers	NCBI Accession No.	BOLD process IDs	Collected from	Phylum	Class	Order	Family	Genus	Species
Morph0001	7	W	MN594583; MN594619; MN594655; MN594691; MN594727	MN447522	SAEVG001-20	Pitfall	Arthropoda	Insecta	Orthoptera	Gryllidae	*Neonemobius*	
Morph0002	100	U		MN447523	SAEVG002-20	Pitfall	Arthropoda	Insecta	Hymenoptera	Formicidae	*Cardiocondyla*	
Morph0004	1	U		MN447524	SAEVG003-20	Pitfall	Arthropoda	Insecta	Diptera	Cecidomyiidae		
Morph0005	7	U		MN447525	SAEVG004-20	Pitfall	Arthropoda	Insecta	Hemiptera	Cydnidae		
Morph0007	1	U		MN447526	SAEVG005-20	Pitfall	Arthropoda	Insecta	Hemiptera	Cicadellidae	*Maiestas*	
Morph0008	62	U		MN447527	SAEVG006-20	Pitfall	Arthropoda	Collembola	Entomobryomorpha	Entomobryidae	*Entomobrya*	*neotenica*
Morph0009	5	W	MN594584; MN594620; MN594656; MN594692; MN594728	MN447528	SAEVG007-20	Pitfall	Arthropoda	Insecta	Hemiptera	Pyrrhocoridae	*Dysdercus*	
Morph0010	1	U		MN447529	SAEVG008-20	Pitfall and leaf litter	Arthropoda	Insecta	Hymenoptera	Formicidae	*Cardiocondyla*	
Morph0011	3	U		MN447530	SAEVG009-20	Pitfall	Arthropoda	Insecta	Coleoptera	Corylophidae	*Sericoderus*	*lateralis*
Morph0012	57	U		MN447531	SAEVG010-20	Pitfall and leaf litter	Arthropoda	Malacostraca	Isopoda	Balloniscidae	*Balloniscus*	*sellowii*
Morph0013	4	U		MN520845	SAEVG011-20	Pitfall and leaf litter	Arthropoda	Arachnida	Araneae	Lycosidae	*Pirata*	
Morph0014	30	U		MN520846	SAEVG012-20	Pitfall and leaf litter	Arthropoda	Collembola	Entomobryomorpha	Paronellidae		
Morph0015	458	U		MN520847	SAEVG013-20	Pitfall and leaf litter	Arthropoda	Collembola	Entomobryomorpha	Entomobryidae	*Homidia*	*similis*
Morph0016	14	A	MN594575	MN520848	SAEVG014-20	Pitfall and leaf litter	Arthropoda	Collembola	Entomobryomorpha	Entomobryidae		
Morph0017	16	U		MN520849	SAEVG015-20	Pitfall	Arthropoda	Collembola	Entomobryomorpha	Entomobryidae	*Entomobrya*	*multifasciata*
Morph0018	47	U		MN520850	SAEVG016-20	Pitfall	Arthropoda	Collembola	Symphypleona	Katiannidae		
Morph0019	5	U		MN520851	SAEVG017-20	Pitfall and leaf litter	Arthropoda	Insecta	Coleoptera	Corylophidae	*Sericoderus*	*lateralis*
Morph0020	3	U		MN520852	SAEVG018-20	Pitfall	Arthropoda	Insecta	Thysanoptera	Phlaeothripidae	*Liothrips*	*infrequens*
Morph0023*	1	W	MN594616; MN594652; MN594688; MN594724; MN594760			Pitfall	Arthropoda	Insecta	Hymenoptera			
Morph0024	62	U		MN520853	SAEVG019-20	Pitfall and leaf litter	Arthropoda	Arachnida	Trombidiformes	Eupodidae		
Morph0025	193	U		MN520854	SAEVG020-20	Pitfall and leaf litter	Arthropoda	Arachnida	Sarcoptiformes	Nanorchestidae	*Nanorchestes*	
Morph0026	5	W	MN594585; MN594621; MN594657; MN594693; MN594729	MN520855	SAEVG021-20	Pitfall	Arthropoda	Insecta	Orthoptera	Gryllidae	*Loxoblemmus*	
Morph0027	7	U		MN520856	SAEVG022-20	Pitfall	Arthropoda	Insecta	Hymenoptera	Formicidae	*Camponotus*	
Morph0028	2	U		MN520857	SAEVG023-20	Pitfall	Arthropoda	Insecta	Hymenoptera	Formicidae	*Pachycondyla*	
Morph0029	1	U		MN520858	SAEVG024-20	Pitfall	Arthropoda	Diplopoda	Polydesmida	Paradoxosomatidae	*Somethus*	
Morph0031	1	U		MN520859	SAEVG025-20	Pitfall	Arthropoda	Insecta	Coleoptera	Staphylinidae	*Tachinus*	
Morph0032	2	U		MN520860	SAEVG026-20	Pitfall	Arthropoda	Insecta	Coleoptera	Carabidae		
Morph0033	26	U		MN520861	SAEVG027-20	Pitfall and leaf litter	Arthropoda	Insecta	Hymenoptera	Formicidae	*Cardiocondyla*	
Morph0034	15	U		MN520862	SAEVG028-20	Pitfall and leaf litter	Arthropoda	Insecta	Hymenoptera	Formicidae	*Meranoplus*	

Morph0035	114	U		MN520863	SAEVG029-20	Pitfall and leaf litter	Arthropoda	Insecta	Hymenoptera	Formicidae		
Morph0036	25	U		MN520864	SAEVG030-20	Pitfall and leaf litter	Arthropoda	Insecta	Hymenoptera	Formicidae	*Crematogaster*	
Morph0037	4	U		MN520865	SAEVG031-20	Pitfall and leaf litter	Arthropoda	Insecta	Hymenoptera	Formicidae	*Nylanderia*	
Morph0038	7	U		MN520866	SAEVG032-20	Pitfall	Arthropoda	Arachnida	Araneae	Lycosidae	*Trochosa*	*aquatica*
Morph0039	3	U		MN520867	SAEVG033-20	Pitfall	Arthropoda	Insecta	Diptera	Anthomyiidae	*Pegomya*	
Morph0040	22	U		MN520868	SAEVG034-20	Pitfall and leaf litter	Arthropoda	Insecta	Diptera	Phoridae		
Morph0041	2	U		MN520869	SAEVG035-20	Pitfall	Arthropoda	Insecta	Dermaptera	Anisolabididae	*Anisolabis*	
Morph0042	14	W*		MN520870	SAEVG036-20	Pitfall and leaf litter	Arthropoda	Insecta	Diptera	Sciaridae	*Cosmosciara*	
Morph0044	21	U		MN520871	SAEVG037-20	Pitfall and leaf litter	Arthropoda	Collembola	Entomobryomorpha	Entomobryidae		
Morph0045	4	C	MN594564	MN520872	SAEVG038-20	Pitfall	Arthropoda	Arachnida	Araneae	Linyphiidae	*Gnathonarium*	
Morph0046	67	U		MN520873	SAEVG039-20	Pitfall and leaf litter	Arthropoda	Collembola	Symphypleona	Katiannidae	*Sminthurinus*	
Morph0047	179	U		MN520874	SAEVG040-20	Pitfall and leaf litter	Arthropoda	Collembola	Entomobryomorpha	Entomobryidae		
Morph0048	35	U		MN520875	SAEVG041-20	Pitfall and leaf litter	Arthropoda	Collembola	Poduromorpha	Neanuridae		
Morph0049	1	U		MN520876	SAEVG042-20	Pitfall	Arthropoda	Insecta	Hymenoptera	Diapriidae	*Basalys*	
Morph0050	1	U		MN520877	SAEVG043-20	Pitfall	Arthropoda	Insecta	Diptera	Phoridae		
Morph0051	7	U		MN520878	SAEVG044-20	Pitfall and leaf litter	Arthropoda	Insecta	Hymenoptera	Encyrtidae	*Pseudleptomastix*	
Morph0052	5	U		MN520879	SAEVG045-20	Pitfall	Arthropoda	Arachnida	Trombidiformes	Anystidae	*Anystis*	*agilis*
Morph0053	30	W*		MN520880	SAEVG046-20	Pitfall and leaf litter	Arthropoda	Arachnida	Sarcoptiformes	Oppiidae	*Berniniella*	*hauseri*
Morph0054	52	U		MN520881	SAEVG047-20	Pitfall and leaf litter	Arthropoda	Arachnida	Sarcoptiformes	Oribatellidae		
Morph0055	3	U		MN520882	SAEVG048-20	Pitfall and leaf litter	Arthropoda	Arachnida	Sarcoptiformes	Scutoverticidae	*Exochocepheus*	
Morph0056	117	U		MN520883	SAEVG049-20	Pitfall and leaf litter	Arthropoda	Insecta	Hemiptera	Pseudococcidae	*Antonina*	*graminis*
Morph0058	1	U		MN520884	SAEVG050-20	Pitfall	Arthropoda	Insecta	Coleoptera	Corylophidae		
Morph0060	12	U		MN520885	SAEVG051-20	Pitfall and leaf litter	Arthropoda	Arachnida	Trombidiformes	Cunaxidae	*Lupaeus*	
Morph0062	4	U		MN520886	SAEVG052-20	Pitfall and leaf litter	Arthropoda	Arachnida	Mesostigmata	Laelapidae		
Morph0063	2	U		MN520887	SAEVG053-20	Pitfall	Arthropoda	Arachnida	Trombidiformes	Raphignathidae		
Morph0068	2	U		MN520888	SAEVG054-20	Pitfall	Arthropoda	Insecta	Hymenoptera	Formicidae	*Tetramorium*	*lanuginosum*
Morph0069	1	U		MN520889	SAEVG055-20	Pitfall	Arthropoda	Insecta	Hymenoptera	Formicidae	*Monomorium*	
Morph0070	76	A	MN594576	MN520890	SAEVG056-20	Pitfall and leaf litter	Arthropoda	Insecta	Hymenoptera	Formicidae	*Technomyrmex*	
Morph0071	2	U		MN520891	SAEVG057-20	Pitfall	Arthropoda	Insecta	Coleoptera	Anthicidae		
Morph0072	11	U		MN520892	SAEVG058-20	Pitfall and leaf litter	Arthropoda	Insecta	Coleoptera	Latridiidae	*Corticarina*	
Morph0073	7	U		MN520893	SAEVG059-20	Pitfall and leaf litter	Arthropoda	Insecta	Coleoptera	Discolomatidae	*Aphanocephalus*	
Morph0074	2	U		MN520894	SAEVG060-20	Pitfall	Arthropoda	Insecta	Coleoptera	Carabidae	*Amara*	*convexior*
Morph0075	3	U		MN520895	SAEVG061-20	Pitfall	Arthropoda	Insecta	Diptera	Drosophilidae	*Zaprionus*	*indianus*
Morph0076	1	W	MN594586; MN594622; MN594658; MN594694; MN594730	MN520896	SAEVG062-20	Pitfall	Arthropoda	Arachnida	Araneae	Phrurolithidae	*Orthobula*	
Morph0077	1	U		MN520897	SAEVG063-20	Pitfall	Arthropoda	Arachnida	Araneae	Araneidae	*Eriovixia*	*excelsa*
Morph0078	4	W*		MN520898	SAEVG064-20	Pitfall	Arthropoda	Arachnida	Araneae	Linyphiidae	*Bathyphantes*	
Morph0079	3	U		MN520899	SAEVG065-20	Pitfall	Arthropoda	Collembola	Symphypleona	Katiannidae		
Morph0080	2	W	MN594587; MN594623; MN594659; MN594695; MN594731	MN520900	SAEVG066-20	Pitfall	Arthropoda	Insecta	Diptera	Phoridae	*Dohrniphora*	
Morph0081	9	W_MULTIPLE		MN520901	SAEVG067-20	Pitfall	Arthropoda	Insecta	Hymenoptera	Platygastridae		
Morph0082	3	W	MN594588; MN594624; MN594660; MN594696; MN594732	MN520902	SAEVG068-20	Pitfall	Arthropoda	Insecta	Hymenoptera	Platygastridae		

Morph0083	3	U		MN520903	SAEVG069-20	Pitfall	Arthropoda	Insecta	Thysanoptera	Phlaeothripidae	*Teuchothrips*	*ater*
Morph0084	2	U		MN520904	SAEVG070-20	Pitfall	Arthropoda	Insecta	Hymenoptera	Platygastridae		
Morph0085	4	W_MULTIPLE+C	MN594565	MN520905	SAEVG071-20	Pitfall	Arthropoda	Insecta	Hymenoptera	Platygastridae		
Morph0086	15	U		MN520906	SAEVG072-20	Pitfall	Arthropoda	Insecta	Hymenoptera	Mymaridae	*Camptoptera*	
Morph0089	2	W*		MN520907	SAEVG073-20	Pitfall and leaf litter	Arthropoda	Insecta	Hymenoptera	Platygastridae	*Fidiobia*	
Morph0092	20	U		MN520908	SAEVG074-20	Pitfall	Arthropoda	Collembola	Symphypleona	Katiannidae	*Sminthurinus*	*elegans*
Morph0095	14	W	MN594589; MN594625; MN594661; MN594697; MN594733	MN520909	SAEVG075-20	Pitfall	Arthropoda	Insecta	Hymenoptera	Formicidae	*Cardiocondyla*	
Morph0096	1	U		MN520910	SAEVG076-20	Pitfall	Arthropoda	Insecta	Hymenoptera	Formicidae	*Cardiocondyla*	
Morph0098	1	W	MN594590; MN594626; MN594662; MN594698; MN594734	MN520911	SAEVG077-20	Pitfall	Arthropoda	Insecta	Hemiptera	Aphididae	*Phorodon*	*humuli*
Morph0099	1	U		MN520912	SAEVG078-20	Pitfall	Arthropoda	Insecta	Orthoptera	Gryllidae	*Cardiodactylus*	
Morph0100	7	U		MN520913	SAEVG079-20	Pitfall and leaf litter	Arthropoda	Insecta	Hymenoptera	Formicidae	*Tapinoma*	*melanocephalum*
Morph0101	1	U		MN520914	SAEVG080-20	Pitfall	Arthropoda	Arachnida	Trombidiformes	Erythraeidae	*Leptus*	
Morph0102	2	U		MN520915	SAEVG081-20	Pitfall	Arthropoda	Arachnida	Trombidiformes	Erythraeidae	*Leptus*	
Morph0103	1	U		MN520916	SAEVG082-20	Pitfall	Arthropoda	Insecta	Hemiptera	Cicadellidae	*Batracomorphus*	
Morph0104	1	U		MN520917	SAEVG083-20	Pitfall	Arthropoda	Insecta	Hemiptera	Cicadellidae	*Osbornellus*	
Morph0106	1	U		MN520918	SAEVG084-20	Pitfall	Arthropoda	Insecta	Hemiptera	Coreidae		
Morph0107	1	U		MN520919	SAEVG085-20	Leaf Litter	Arthropoda	Insecta	Diptera	Muscidae	*Lispe*	
Morph0108	1	U		MN520920	SAEVG086-20	Leaf Litter	Arthropoda	Insecta	Diptera	Muscidae	*Lispe*	
Morph0109	1	U		MN520921	SAEVG087-20	Leaf Litter	Arthropoda	Insecta	Diptera	Sepsidae	*Sepsis*	
Morph0110	2	U		MN520922	SAEVG088-20	Leaf Litter	Arthropoda	Insecta	Hemiptera	Delphacidae	*Nilaparvata*	*lugens*
Morph0111	10	W	MN594591; MN594627; MN594663; MN594699; MN594735	MN520923	SAEVG089-20	Leaf Litter	Arthropoda	Insecta	Hemiptera	Delphacidae	*Nilaparvata*	*lugens*
Morph0112	7	U		MN520924	SAEVG090-20	Leaf Litter	Arthropoda	Insecta	Hymenoptera	Ichneumonidae	*Megastylus*	
Morph0113	1	U		MN520925	SAEVG091-20	Leaf Litter	Arthropoda	Insecta	Hymenoptera	Formicidae	*Tetramorium*	*lanuginosum*
Morph0114	1	U		MN520926	SAEVG092-20	Leaf Litter	Arthropoda	Insecta	Diptera	Ceratopogonidae		
Morph0115	1	U		MN520927	SAEVG093-20	Leaf Litter	Arthropoda	Insecta	Diptera	Chironomidae	*Smittia*	
Morph0116	1	U		MN520928	SAEVG094-20	Leaf Litter	Arthropoda	Insecta	Hemiptera	Cicadellidae	*Empoasca*	
Morph0117	9	U		MN520929	SAEVG095-20	Leaf Litter	Arthropoda	Collembola	Entomobryomorpha	Isotomidae	*Isotomodes*	
Morph0118	11	U		MN520930	SAEVG096-20	Pitfall and leaf litter	Arthropoda	Insecta	Psocodea	Liposcelididae	*Liposcelis*	
Morph0121	2	U		MN520931	SAEVG097-20	Leaf Litter	Arthropoda	Insecta	Hemiptera	Cicadellidae		
Morph0122	3	U		MN520932	SAEVG098-20	Pitfall and leaf litter	Arthropoda	Insecta	Diptera	Phoridae	*Megaselia*	*rufipes*
Morph0123	2	U		MN520933	SAEVG099-20	Leaf Litter	Arthropoda	Insecta	Diptera	Phoridae		
Morph0124	2	U		MN520934	SAEVG100-20	Leaf Litter	Arthropoda	Insecta	Diptera	Ephydridae	*Lamproscatella*	
Morph0125	1	U		MN520935	SAEVG101-20	Leaf Litter	Arthropoda	Insecta	Diptera	Hybotidae		
Morph0126	1	W*		MN520936	SAEVG102-20	Leaf Litter	Arthropoda	Insecta	Diptera	Dolichopodidae	*Syntormon*	
Morph0127	7	U		MN520937	SAEVG103-20	Leaf Litter	Arthropoda	Diplura	Dicellurata	Japygidae	*Parajapyx*	
Morph0128	9	U		MN520938	SAEVG104-20	Leaf Litter	Arthropoda	Insecta	Hymenoptera	Formicidae	*Carebara*	
Morph0129	2	U		MN520939	SAEVG105-20	Leaf Litter	Arthropoda	Insecta	Hymenoptera	Formicidae	*Cardiocondyla*	
Morph0130	2	U		MN520940	SAEVG106-20	Pitfall and leaf litter	Arthropoda	Insecta	Hymenoptera	Eulophidae	*Aulogymnus*	
Morph0131	2	U		MN520941	SAEVG107-20	Leaf Litter	Arthropoda	Collembola	Symphypleona	Katiannidae	*Sminthurinus*	*elegans*
Morph0133	17	U		MN520942	SAEVG108-20	Pitfall and leaf litter	Arthropoda	Arachnida	Sarcoptiformes	Chamobatidae	*Chamobates*	

Morph0135	1	U		MN520943	SAEVG109-20	Leaf Litter	Arthropoda	Arachnida	Sarcoptiformes	Chamobatidae	*Chamobates*	
Morph0137	3	U		MN520944	SAEVG110-20	Leaf Litter	Arthropoda	Arachnida	Mesostigmata	Blattisociidae		
Morph0142	12	U		MN520945	SAEVG111-20	Leaf Litter	Arthropoda	Collembola	Entomobryomorpha	Entomobryidae	*Entomobrya*	
Morph0146	29	U		MN520946	SAEVG112-20	Pitfall and leaf litter	Arthropoda	Collembola	Entomobryomorpha	Entomobryidae	*Willowsia*	
Morph0148	2	W	MN594592; MN594628; MN594664; MN594700; MN594736	MN520947	SAEVG113-20	Pitfall	Arthropoda	Arachnida	Araneae	Gnaphosidae	*Zelotes*	
Morph0149	5	U		MN520948	SAEVG114-20	Pitfall	Arthropoda	Insecta	Hymenoptera	Formicidae	*Camponotus*	
Morph0150	1	U		MN520949	SAEVG115-20	Pitfall	Arthropoda	Insecta	Orthoptera	Gryllidae	*Gryllus/Teleogryllus*	
Morph0151	19	U		MN520950	SAEVG116-20	Pitfall	Arthropoda	Insecta	Hemiptera	Aphididae	*Hysteroneura*	*setariae*
Morph0152	2	W+C	MN594593; MN594629; MN594665; MN594701; MN594737+MN594566	MN520951	SAEVG117-20	Pitfall	Arthropoda	Insecta	Hymenoptera	Platygastridae	*Dicroscelio* sp.	
Morph0155	1	U		MN520952	SAEVG118-20	Pitfall	Arthropoda	Arachnida	Ixodida	Ixodidae	*Haemaphysalis*	
Morph0156	9	U		MN520953	SAEVG119-20	Pitfall and leaf litter	Arthropoda	Insecta	Lepidoptera	Cosmopterigidae		
Morph0161	2	U		MN520954	SAEVG120-20	Pitfall	Arthropoda	Arachnida	Araneae	Oxyopidae	*Oxyopes*	
Morph0162	1	U		MN520955	SAEVG121-20	Pitfall	Arthropoda	Arachnida	Araneae	Selenopidae	*Selenops*	
Morph0164	2	U		MN520956	SAEVG122-20	Pitfall	Arthropoda	Insecta	Diptera	Phoridae	*Megaselia*	
Morph0165	2	U		MN520957	SAEVG123-20	Pitfall	Arthropoda	Insecta	Diptera	Phoridae	*Megaselia*	
Morph0167	9	A	MN594577	MN520958	SAEVG124-20	Pitfall	Arthropoda	Insecta	Diptera	Drosophilidae	*Drosophila*	*jambulina*
Morph0168	2	U		MN520959	SAEVG125-20	Pitfall	Arthropoda	Arachnida	Araneae	Theridiidae	*Coleosoma*	
Morph0169	7	U		MN520960	SAEVG126-20	Pitfall and leaf litter	Arthropoda	Arachnida	Araneae	Theridiidae	*Coleosoma*	
Morph0170	1	U		MN520961	SAEVG127-20	Pitfall	Arthropoda	Insecta	Hymenoptera	Formicidae		
Morph0171	5	W + C	MN594594; MN594630; MN594666; MN594702; MN594738+MN594567	MN520962	SAEVG128-20	Pitfall	Arthropoda	Insecta	Hymenoptera	Diapriidae	*Trichopria* sp.	
Morph0173	63	U		MN520963	SAEVG129-20	Pitfall	Arthropoda	Collembola	Poduromorpha	Hypogastruridae		
Morph0175	1	U		MN520964	SAEVG130-20	Pitfall	Arthropoda	Insecta	Hemiptera	Cicadellidae	*Batracomorphus*	
Morph0178	3	U		MN520965	SAEVG131-20	Pitfall and leaf litter	Arthropoda	Arachnida	Sarcoptiformes	Scheloribatidae	*Scheloribates*	
Morph0179	3	U		MN520966	SAEVG132-20	Pitfall and leaf litter	Arthropoda	Insecta	Diptera	Psychodidae	*Psychoda*	
Morph0180	2	W*		MN520967	SAEVG133-20	Leaf Litter	Arthropoda	Insecta	Diptera	Sciaridae		
Morph0181	1	U		MN520968	SAEVG134-20	Leaf Litter	Arthropoda	Insecta	Diptera	Ceratopogonidae		
Morph0182	1	W	MN594595; MN594631; MN594667; MN594703; MN594739	MN520969	SAEVG135-20	Leaf Litter	Arthropoda	Insecta	Hymenoptera	Platygastridae	*Idris* sp.	
Morph0186	1	U		MN520970	SAEVG136-20	Leaf Litter	Arthropoda	Insecta	Blattodea	Blaberidae	*Pycnoscelus*	*surinamensis*
Morph0187	1	U		MN520971	SAEVG137-20	Leaf Litter	Arthropoda	Insecta	Coleoptera	Scirtidae		
Morph0188	1	U		MN520972	SAEVG138-20	Leaf Litter	Arthropoda	Insecta	Coleoptera	Staphylinidae	*Philonthus*	
Morph0189	2	W	MN594596; MN594632; MN594668; MN594704; MN594740	MN520973	SAEVG139-20	Leaf Litter	Arthropoda	Insecta	Hymenoptera	Bethylidae	*Laelius*	
Morph0191	1	U		MN520974	SAEVG140-20	Leaf Litter	Arthropoda	Insecta	Diptera	Psychodidae	*Sergentomyia*	*babu*
Morph0192	2	W*		MN520975	SAEVG141-20	Leaf Litter	Arthropoda	Insecta	Diptera	Sciaridae		
Morph0193	6	U		MN520976	SAEVG142-20	Pitfall and leaf litter	Arthropoda	Insecta	Diptera	Ephydridae	*Scatella*	
Morph0194	3	U		MN520977	SAEVG143-20	Leaf Litter	Arthropoda	Insecta	Diptera	Sphaeroceridae	*Spelobia*	
Morph0195	1	U		MN520978	SAEVG144-20	Leaf Litter	Arthropoda	Insecta	Coleoptera	Coccinellidae	*Scymnus*	*nubilus*
Morph0196	20	C	MN594568	MN520979	SAEVG145-20	Leaf Litter	Arthropoda	Arachnida	Mesostigmata	Urodinychidae	*Uroobovella*	*dryocoetes*
Morph0197	3	U		MN520980	SAEVG146-20	Leaf Litter	Arthropoda	Arachnida	Mesostigmata	Urodinychidae	*Uroobovella*	*dryocoetes*
Morph0198	6	U		MN520981	SAEVG147-20	Leaf Litter	Arthropoda	Collembola				
Morph0201	1	U		MN520982	SAEVG148-20	Pitfall	Arthropoda	Insecta	Coleoptera	Scarabaeidae	*Onthophagus*	

Morph0202	1	U		MN520983	SAEVG149-20	Pitfall	Arthropoda	Insecta	Coleoptera	Curculionidae	*Pachytychius*	
Morph0203	4	U		MN520984	SAEVG150-20	Pitfall	Arthropoda	Insecta	Blattodea	Ectobiidae		
Morph0204	1	U		MN520985	SAEVG151-20	Pitfall	Arthropoda	Insecta	Diptera	Muscidae	*Atherigona*	*orientalis*
Morph0205	2	U		MN520986	SAEVG152-20	Pitfall and leaf litter	Arthropoda	Arachnida	Araneae	Philodromidae	*Thanatus*	*ketani*
Morph0206	165	W	MN594597; MN594633; MN594669; MN594705; MN594741	MN520987	SAEVG153-20	Pitfall	Arthropoda	Insecta	Hymenoptera	Formicidae	*Paratrechina*	*longicornis*
Morph0207	97	U		MN520988	SAEVG154-20	Pitfall	Arthropoda	Insecta	Hymenoptera	Formicidae	*Crematogaster*	
Morph0209	1	U		MN520989	SAEVG155-20	Pitfall	Arthropoda	Insecta	Hymenoptera	Formicidae		
Morph0210	1	W	MN594598; MN594634; MN594670; MN594706; MN594742	MN520990	SAEVG156-20	Pitfall	Arthropoda	Insecta	Coleoptera	Chrysomelidae	*Bruchus*	
Morph0211	2	U		MN520991	SAEVG157-20	Pitfall	Arthropoda	Arachnida	Araneae	Theridiidae	*Coleosoma*	
Morph0212	1	U		MN520992	SAEVG158-20	Pitfall	Arthropoda	Arachnida	Araneae	Theridiidae	*Steatoda*	*cingulata*
Morph0213	7	W	MN594599; MN594635; MN594671; MN594707; MN594743	MN520993	SAEVG159-20	Pitfall and leaf litter	Arthropoda	Insecta	Hemiptera	Psyllidae	*Heteropsylla*	*cubana*
Morph0214	14	W	MN594600; MN594636; MN594672; MN594708; MN594744	MN520994	SAEVG160-20	Pitfall	Arthropoda	Insecta	Hemiptera	Delphacidae	*Muellerianella*	
Morph0215	2	U		MN520995	SAEVG161-20	Pitfall	Arthropoda	Insecta	Coleoptera	Archeocrypticidae		
Morph0216	2	U		MN520996	SAEVG162-20	Pitfall	Arthropoda	Insecta	Diptera	Cecidomyiidae		
Morph0217	2	U		MN520997	SAEVG163-20	Pitfall	Arthropoda	Insecta	Coleoptera	Staphylinidae	*Tachinus*	
Morph0218	1	U		MN520998	SAEVG164-20	Pitfall	Arthropoda	Insecta	Coleoptera	Staphylinidae	*Aleochara*	
Morph0219	3	U		MN520999	SAEVG165-20	Pitfall and leaf litter	Arthropoda	Insecta	Hemiptera	Cydnidae	*Macroscytus*	
Morph0220	7	W	MN594601; MN594637; MN594673; MN594709; MN594745	MN521000	SAEVG166-20	Pitfall	Arthropoda	Insecta	Hemiptera	Delphacidae		
Morph0222	1	U		MN521001	SAEVG167-20	Pitfall	Arthropoda	Insecta	Hemiptera	Cicadellidae	*Cicadella*	
Morph0223	2	U		MN521002	SAEVG168-20	Pitfall	Arthropoda	Insecta	Orthoptera	Gryllidae	*Teleogryllus*	*infernalis*
Morph0224	1	U		MN521003	SAEVG169-20	Pitfall	Arthropoda	Insecta	Psocodea	Liposcelididae		
Morph0225	2	U		MN521004	SAEVG170-20	Pitfall and leaf litter	Arthropoda	Insecta	Diptera	Sciaridae		
Morph0226	1	U		MN521005	SAEVG171-20	Pitfall	Arthropoda	Insecta	Diptera	Phoridae		
Morph0227	6	U		MN521006	SAEVG172-20	Pitfall	Arthropoda	Collembola	Entomobryomorpha	Entomobryidae	*Homidia*	*socia*
Morph0228	32	U		MN521007	SAEVG173-20	Pitfall and leaf litter	Arthropoda	Collembola	Entomobryomorpha	Entomobryidae	*Entomobrya*	*assuta*
Morph0229	6	U		MN521008	SAEVG174-20	Pitfall	Arthropoda	Collembola	Entomobryomorpha	Entomobryidae	*Entomobrya*	*multifasciata*
Morph0230	45	U		MN521009	SAEVG175-20	Pitfall and leaf litter	Arthropoda	Collembola	Entomobryomorpha	Entomobryidae		
Morph0232	2	U		MN521010	SAEVG176-20	Pitfall	Arthropoda	Insecta	Coleoptera	Staphylinidae		
Morph0233	1	U		MN521011	SAEVG177-20	Pitfall	Arthropoda	Insecta	Coleoptera	Staphylinidae	*Oxytelus*	
Morph0234	1	U		MN521012	SAEVG178-20	Pitfall	Arthropoda	Insecta	Hymenoptera	Formicidae	*Camponotus*	
Morph0235	1	U		MN521013	SAEVG179-20	Pitfall	Arthropoda	Arachnida	Araneae	Lycosidae	*Hogna*	
Morph0236	1	U		MN521014	SAEVG180-20	Pitfall	Arthropoda	Arachnida	Araneae	Oxyopidae	*Oxyopes*	
Morph0237	1	U		MN521015	SAEVG181-20	Pitfall	Arthropoda	Arachnida	Araneae	Philodromidae	*Philodromus*	
Morph0239	18	U		MN521016	SAEVG182-20	Pitfall	Arthropoda	Insecta	Hymenoptera	Formicidae	*Pheidole*	
Morph0241	5	U		MN521017	SAEVG183-20	Pitfall	Arthropoda	Insecta	Coleoptera	Mycteridae		
Morph0242	1	U		MN521018	SAEVG184-20	Pitfall	Arthropoda	Insecta	Coleoptera	Curculionidae		
Morph0244	4	U		MN521019	SAEVG185-20	Pitfall	Arthropoda	Insecta	Hemiptera	Aphididae	*Schizaphis*	
Morph0245	3	U		MN521020	SAEVG186-20	Pitfall	Arthropoda	Insecta	Hemiptera	Aphididae	*Hysteroneura*	*setariae*
Morph0246	1	U		MN521021	SAEVG187-20	Pitfall	Arthropoda	Insecta	Thysanoptera	Phlaeothripidae	*Haplothrips*	*ganglbaueri*
Morph0248	145	C	MN594569	MN521022	SAEVG188-20	Pitfall and leaf litter	Arthropoda	Collembola	Entomobryomorpha	Entomobryidae	*Entomobrya*	
Morph0249	6	U		MN521023	SAEVG189-20	Pitfall	Arthropoda	Collembola	Entomobryomorpha	Entomobryidae	*Seira*	*delamarei*

Morph0250	2	U		MN521024	SAEVG190-20	Pitfall	Arthropoda	Insecta	Diptera	Ceratopogonidae		
Morph0251	1	U		MN521025	SAEVG191-20	Pitfall	Arthropoda	Insecta	Hymenoptera	Encyrtidae	*Ooencyrtus*	
Morph0252	1	U		MN521026	SAEVG192-20	Pitfall	Arthropoda	Insecta	Diptera	Sciaridae		
Morph0255	1	U		MN521027	SAEVG193-20	Leaf Litter	Arthropoda	Insecta	Thysanoptera	Phlaeothripidae		
Morph0256*	1	C	MN594570			Leaf Litter	Arthropoda	Arachnida	Araneae			
Morph0257	1	U		MN521027	SAEVG194-20	Leaf Litter	Arthropoda	Insecta	Hymenoptera	Bethylidae	*Holepyris*	*atamensis*
Morph0258	1	U		MN521028	SAEVG195-20	Leaf Litter	Arthropoda	Insecta	Coleoptera	Latridiidae	*Corticarina*	
Morph0259	2	U		MN521029	SAEVG196-20	Leaf Litter	Arthropoda	Insecta	Hemiptera	Enicocephalidae		
Morph0260	1	A	MN594578	MN521030	SAEVG197-20	Leaf Litter	Arthropoda	Insecta	Hemiptera	Reduviidae		
Morph0261	8	U		MN521031	SAEVG198-20	Pitfall and leaf litter	Arthropoda	Insecta	Diptera	Sciaridae		
Morph0262	7	U		MN521033	SAEVG199-20	Leaf Litter	Arthropoda	Insecta	Coleoptera	Elateridae	*Hemicrepidius*	
Morph0264	1	U		MN521034	SAEVG200-20	Leaf Litter	Arthropoda	Insecta	Coleoptera	Carabidae	*Microlestes*	*minutulus*
Morph0265	1	U		MN521035	SAEVG201-20	Pitfall	Arthropoda	Insecta	Hemiptera	Reduviidae	*Peirates*	
Morph0267	4	U		MN521036	SAEVG202-20	Pitfall	Arthropoda	Chilopoda	Lithobiomorpha	Lithobiidae	*Lithobius*	
Morph0268	1	U		MN521037	SAEVG203-20	Pitfall	Arthropoda	Insecta	Orthoptera	Acrididae	*Eyprepocnemis*	
Morph0269	2	W	MN594602; MN594638; MN594674; MN594710; MN594746	MN521038	SAEVG204-20	Pitfall	Arthropoda	Insecta	Hymenoptera	Platygastridae	*Scelio* sp.	
Morph0271	3	U		MN521039	SAEVG205-20	Pitfall and leaf litter	Arthropoda	Arachnida	Araneae	Linyphiidae	*Agyneta*	
Morph0272	1	U		MN521040	SAEVG206-20	Pitfall	Arthropoda	Insecta	Lepidoptera	Cosmopterigidae		
Morph0273	1	U		MN521041	SAEVG207-20	Pitfall	Arthropoda	Arachnida	Araneae	Oxyopidae	*Oxyopes*	
Morph0274	1	U		MN521042	SAEVG208-20	Pitfall	Arthropoda	Arachnida	Araneae	Thomisidae		
Morph0275	1	U		MN521043	SAEVG209-20	Pitfall	Arthropoda	Arachnida	Trombidiformes	Erythraeidae	*Leptus*	
Morph0276	1	U		MN521044	SAEVG210-20	Pitfall	Arthropoda	Arachnida	Trombidiformes	Erythraeidae	*Leptus*	
Morph0277	6	U		MN521045	SAEVG211-20	Pitfall	Arthropoda	Insecta	Hymenoptera	Diapriidae		
Morph0278	5	U		MN521046	SAEVG212-20	Pitfall and leaf litter	Arthropoda	Insecta	Hymenoptera	Formicidae	*Paratrechina*	
Morph0280	1	U		MN521047	SAEVG213-20	Pitfall and leaf litter	Arthropoda	Insecta	Zygentoma	Nicoletiidae	*Atelura*	
Morph0281	1	U		MN521048	SAEVG214-20	Pitfall	Arthropoda	Insecta	Coleoptera	Erotylidae	*Cryptophilus*	
Morph0282	1	U		MN521049	SAEVG215-20	Pitfall	Arthropoda	Insecta	Hemiptera	Lygaeidae	*Spilostethus*	
Morph0283	1	U		MN521050	SAEVG216-20	Pitfall	Arthropoda	Insecta	Hemiptera	Miridae	*Pilophorus*	*typicus*
Morph0285	4	W	MN594603; MN594639; MN594675; MN594711; MN594747	MN521051	SAEVG217-20	Pitfall and leaf litter	Arthropoda	Insecta	Diptera	Phoridae		
Morph0287	1	U		MN521052	SAEVG218-20	Pitfall	Arthropoda	Arachnida	Ixodida	Ixodidae	*Hyalomma*	*detritum*
Morph0288*	1	W	MN594617; MN594653; MN594689; MN594725; MN594761			Pitfall	Arthropoda	Insecta	Hemiptera			
Morph0289	1	U		MN521053	SAEVG219-20	Pitfall	Arthropoda	Insecta	Hemiptera	Aphididae	*Aphis*	
Morph0290	1	U		MN521054	SAEVG220-20	Pitfall	Arthropoda	Insecta	Diptera	Sphaeroceridae		
Morph0291*	1	A	MN594579			Pitfall	Arthropoda	Insecta	Thysanoptera			
Morph0293	2	W	MN594604; MN594640; MN594676; MN594712; MN594748	MN521055	SAEVG221-20	Pitfall	Arthropoda	Insecta	Hymenoptera	Platygastridae	*Telenomus*	
Morph0294	7	W+**A**	MN594605; MN594641; MN594677; MN594713; MN594749+**MN594580**	MN521056	SAEVG222-20	Pitfall and leaf litter	Arthropoda	Insecta	Hymenoptera	Platygastridae		
Morph0295*	1	C	MN594571			Pitfall	Arthropoda	Insecta	Hymenoptera			
Morph0297	1	U		MN521057	SAEVG223-20	Pitfall	Arthropoda	Insecta	Hemiptera	Coreidae	*Cletus*	*bipunctatus*
Morph0298	1	U		MN521058	SAEVG224-20	Pitfall	Arthropoda	Insecta	Hemiptera	Reduviidae	*Peirates*	

Morph0299	2	U		MN521059	SAEVG225-20	Pitfall and leaf litter	Arthropoda	Insecta	Hemiptera	Reduviidae	*Sastrapada*	
Morph0300	1	U		MN521060	SAEVG226-20	Pitfall	Arthropoda	Arachnida	Araneae	Gnaphosidae	*Trachyzelotes*	
Morph0301	1	U		MN521061	SAEVG227-20	Pitfall	Arthropoda	Arachnida	Araneae	Thomisidae	*Xysticus*	*ulmi*
Morph0303	1	U		MN521062	SAEVG228-20	Pitfall	Arthropoda	Arachnida	Araneae	Zodariidae	*Zodarion*	
Morph0304	2	U		MN521063	SAEVG229-20	Pitfall	Arthropoda	Insecta	Blattodea	Ectobiidae	*Blattella*	*biligata*
Morph0305	1	U		MN521064	SAEVG230-20	Pitfall	Arthropoda	Arachnida	Araneae	Lycosidae	*Zoica*	
Morph0306	1	C	MN594572	MN521065	SAEVG231-20	Pitfall	Arthropoda	Arachnida	Araneae	Linyphiidae	*Bathyphantes*	*approximatus*
Morph0307	1	U		MN521066	SAEVG232-20	Pitfall	Arthropoda	Insecta	Coleoptera	Tenebrionidae	*Uloma*	
Morph0308	1	U		MN521067	SAEVG233-20	Pitfall	Arthropoda	Insecta	Coleoptera	Curculionidae	*Melanterius*	
Morph0309	1	U		MN521068	SAEVG234-20	Pitfall	Arthropoda	Insecta	Coleoptera	Staphylinidae	*Nudobius*	
Morph0310	1	U		MN521069	SAEVG235-20	Pitfall	Arthropoda	Insecta	Coleoptera	Staphylinidae		
Morph0311	1	U		MN521070	SAEVG236-20	Pitfall	Arthropoda	Insecta	Coleoptera	Carabidae	*Syntomus*	*foveatus*
Morph0312	1	U		MN521071	SAEVG237-20	Pitfall	Arthropoda	Insecta	Hymenoptera	Formicidae	*Myrmicaria*	
Morph0313	5	U		MN521072	SAEVG238-20	Pitfall and leaf litter	Arthropoda	Insecta	Hymenoptera	Formicidae	*Brachyponera*	
Morph0314	1	U		MN521073	SAEVG239-20	Pitfall	Arthropoda	Insecta	Hymenoptera	Formicidae	*Pheidole*	
Morph0315	3	U		MN521074	SAEVG240-20	Pitfall and leaf litter	Arthropoda	Insecta	Hymenoptera	Formicidae	*Cardiocondyla*	*mauritanica*
Morph0316	1	U		MN521075	SAEVG241-20	Pitfall	Arthropoda	Insecta	Hymenoptera	Formicidae	*Monomorium*	
Morph0317	1	U		MN521076	SAEVG242-20	Pitfall	Arthropoda	Insecta	Diptera	Phoridae		
Morph0318	1	U		MN521077	SAEVG243-20	Pitfall	Arthropoda	Insecta	Diptera	Phoridae		
Morph0319	1	U		MN521078	SAEVG244-20	Pitfall	Arthropoda	Arachnida	Araneae	Linyphiidae	*Bathyphantes*	*approximatus*
Morph0320	1	U		MN521079	SAEVG245-20	Pitfall	Arthropoda	Insecta	Hymenoptera	Diapriidae		
Morph0321	2	U		MN521080	SAEVG246-20	Pitfall	Arthropoda	Insecta	Hymenoptera	Diapriidae		
Morph0322	1	U		MN521081	SAEVG247-20	Pitfall	Arthropoda	Insecta	Hymenoptera	Platygastridae		
Morph0324	12	W	MN594606; MN594642; MN594678; MN594714; MN594750	MN521082	SAEVG248-20	Pitfall and leaf litter	Arthropoda	Insecta	Hymenoptera	Platygastridae		
Morph0325	1	U		MN521083	SAEVG249-20	Pitfall	Arthropoda	Insecta	Coleoptera	Cryptophagidae	*Atomaria*	
Morph0326	2	U		MN521084	SAEVG250-20	Pitfall	Arthropoda	Insecta	Hemiptera	Cicadellidae		
Morph0327	1	U		MN521085	SAEVG251-20	Pitfall	Arthropoda	Insecta	Diptera	Cecidomyiidae	*Orseolia*	
Morph0328	1	C+**A**	MN594573+**MN594581**	MN521086	SAEVG252-20	Pitfall	Arthropoda	Insecta	Psocodea	Liposcelididae	*Embidopsocus*	
Morph0329	1	W+**A**	MN594607; MN594643; MN594679; MN594715; MN594751+**MN594582**	MN521087	SAEVG253-20	Pitfall	Arthropoda	Insecta	Hemiptera	Cicadellidae	*Balclutha*	
Morph0330	1	W	MN594608; MN594644; MN594680; MN594716; MN594752	MN521088	SAEVG254-20	Pitfall	Arthropoda	Arachnida	Araneae	Uloboridae	*Uloborus*	*plumipes*
Morph0333	1	U		MN521089	SAEVG255-20	Leaf Litter	Arthropoda	Insecta	Lepidoptera			
Morph0334	1	U		MN521090	SAEVG256-20	Leaf Litter	Arthropoda	Insecta	Coleoptera	Cryptophagidae	*Cryptophagus*	
Morph0335*	1	C	MN594574			Leaf Litter	Arthropoda	Arachnida	Araneae			
Morph0336	1	U		MN521091	SAEVG257-20	Leaf Litter	Arthropoda	Insecta	Coleoptera	Coccinellidae	*Propylaea*	*quatuordecim punctata*
Morph0339	4	U		MN521092	SAEVG258-20	Pitfall and leaf litter	Arthropoda	Insecta	Hymenoptera	Platygastridae		
Morph0342	4	U		MN521093	SAEVG259-20	Pitfall and leaf litter	Arthropoda	Insecta	Thysanoptera	Thripidae	*Scirtothrips*	*dorsalis*
Morph0343*	1	W	MN594618; MN594654; MN594690; MN594726; MN594762			Leaf Litter	Arthropoda	Insecta	Hymenoptera			
Morph0344	1	U		MN521094	SAEVG260-20	Leaf Litter	Arthropoda	Insecta	Diptera	Phoridae		
Morph0345	1	U		MN521095	SAEVG261-20	Leaf Litter	Arthropoda	Insecta	Diptera	Phoridae	*Megaselia*	
Morph0346	1	U		MN521096	SAEVG262-20	Leaf Litter	Arthropoda	Insecta	Diptera	Chironomidae		

Morph0347	1	U		MN521097	SAEVG263-20	Leaf Litter	Arthropoda	Insecta	Hymenoptera	Formicidae	*Strumigenys*	
Morph0348	1	W	MN594609; MN594645; MN594681; MN594717; MN594753	MN521098	SAEVG264-20	Leaf Litter	Arthropoda	Insecta	Coleoptera	Chrysomelidae		
Morph0349	1	U		MN521099	SAEVG265-20	Leaf Litter	Arthropoda	Insecta	Coleoptera	Staphylinidae		
Morph0350	1	U		MN521100	SAEVG266-20	Leaf Litter	Arthropoda	Insecta	Coleoptera	Carabidae		
Morph0352	2	W	MN594610; MN594646; MN594682; MN594718; MN594754	MN521101	SAEVG267-20	Leaf Litter	Arthropoda	Insecta	Coleoptera	Corylophidae		
Morph0354	1	U		MN521102	SAEVG268-20	Leaf Litter	Arthropoda	Insecta	Coleoptera	Staphylinidae		
Morph0355	1	U		MN521103	SAEVG269-20	Leaf Litter	Arthropoda	Insecta	Coleoptera	Staphylinidae	*Atheta*	*brunneipennis*
Morph0356	1	U		MN521104	SAEVG270-20	Leaf Litter	Arthropoda	Diplura	Rhabdura	Campodeidae	*Lepidocampa*	
Morph0357	1	U		MN521105	SAEVG271-20	Leaf Litter	Arthropoda	Arachnida	Araneae	Anapidae	*Anapis*	
Morph0358	1	U		MN521106	SAEVG272-20	Leaf Litter	Arthropoda	Arachnida	Araneae	Anapidae	*Anapis*	
Morph0361	20	U		MN521107	SAEVG273-20	Pitfall and leaf litter	Arthropoda	Collembola	Entomobryomorpha	Isotomidae	*Folsomia*	*quadrioculata*
Morph0363	7	U		MN521108	SAEVG274-20	Leaf Litter	Arthropoda	Collembola	Entomobryomorpha	Isotomidae	*Isotomodes*	
Morph0364	1	U		MN521109	SAEVG275-20	Leaf Litter	Arthropoda	Insecta	Coleoptera	Chrysomelidae	*Chaetocnema*	
Morph0365	2	U		MN521110	SAEVG276-20	Leaf Litter	Arthropoda	Arachnida	Araneae	Salticidae	*Icius*	
Morph0366	1	U		MN521111	SAEVG277-20	Leaf Litter	Arthropoda	Insecta	Diptera	Cecidomyiidae		
Morph0367	1	U		MN521112	SAEVG278-20	Leaf Litter	Arthropoda	Collembola	Entomobryomorpha	Entomobryidae		
Morph0369	1	U		MN521113	SAEVG279-20	Leaf Litter	Arthropoda	Arachnida	Araneae	Oxyopidae	*Oxyopes*	
Morph0370	1	U		MN521114	SAEVG280-20	Leaf Litter	Arthropoda	Insecta	Coleoptera	Corylophidae	*Gloeosoma*	
Morph0371	1	U		MN521115	SAEVG281-20	Leaf Litter	Arthropoda	Insecta	Coleoptera			
Morph0372	2	U		MN521116	SAEVG282-20	Leaf Litter	Arthropoda	Insecta	Coleoptera	Anthicidae		
Morph0373	2	U		MN521117	SAEVG283-20	Pitfall and leaf litter	Arthropoda	Insecta	Thysanoptera	Phlaeothripidae		
Morph0375	1	W	MN594611; MN594647; MN594683; MN594719; MN594755	MN521118	SAEVG284-20	Leaf Litter	Arthropoda	Insecta	Coleoptera	Chrysomelidae	*Monolepta*	
Morph0376	3	W	MN594612; MN594648; MN594684; MN594720; MN594756	MN521119	SAEVG285-20	Leaf Litter	Arthropoda	Insecta	Hymenoptera	Formicidae	*Pheidole*	
Morph0377	1	U		MN521120	SAEVG286-20	Leaf Litter	Arthropoda	Insecta	Hymenoptera	Formicidae	*Pheidole*	
Morph0378	1	U		MN521121	SAEVG287-20	Leaf Litter	Arthropoda	Insecta	Hymenoptera	Formicidae	*Technomyrmex*	
Morph0379	1	U		MN521122	SAEVG288-20	Leaf Litter	Arthropoda	Insecta	Hemiptera	Tingidae	*Teleonemia*	*scrupulosa*
Morph0380	1	U		MN521123	SAEVG289-20	Leaf Litter	Arthropoda	Insecta	Hemiptera	Lygaeidae	*Nysius*	
Morph0381	1	W	MN594613; MN594649; MN594685; MN594721; MN594757	MN521124	SAEVG290-20	Leaf Litter	Arthropoda	Insecta	Hemiptera	Cicadellidae	*Agalliopsis*	
Morph0382	3	U		MN521125	SAEVG291-20	Leaf Litter	Arthropoda	Arachnida	Araneae	Linyphiidae	*Bathyphantes*	
Morph0385	1	U		MN521126	SAEVG292-20	Leaf Litter	Arthropoda	Insecta	Diptera	Phoridae	*Megaselia*	
Morph0386	1	W	MN594614; MN594650; MN594686; MN594722; MN594758	MN521127	SAEVG293-20	Leaf Litter	Arthropoda	Insecta	Hemiptera	Cicadellidae		
Morph0387	1	U		MN521128	SAEVG294-20	Leaf Litter	Arthropoda	Insecta	Hemiptera	Cicadellidae		
Morph0388	4	U		MN521129	SAEVG295-20	Leaf Litter	Arthropoda	Insecta	Thysanoptera	Thripidae	*Scirtothrips*	*oligochaetus*
Morph0389	1	U		MN521130	SAEVG296-20	Leaf Litter	Arthropoda	Insecta	Hemiptera	Cicadellidae		
Morph0390	1	U		MN521131	SAEVG297-20	Leaf Litter	Arthropoda	Insecta	Lepidoptera	Noctuidae	*Diarsia*	
Morph0391	1	U		MN521132	SAEVG298-20	Leaf Litter	Arthropoda	Insecta	Hymenoptera	Ceraphronidae		
Morph0392	1	U		MN521133	SAEVG299-20	Leaf Litter	Arthropoda	Insecta	Hymenoptera	Formicidae	*Polyrhachis*	
Morph0393	2	W*		MN521134	SAEVG300-20	Leaf Litter	Arthropoda	Insecta	Hymenoptera	Formicidae	*Cardiocondyla*	
Morph0394	1	U		MN521135	SAEVG301-20	Leaf Litter	Arthropoda	Insecta	Hymenoptera	Formicidae	*Pheidole*	

Morph0395	1	U		MN521136	SAEVG302-20	Leaf Litter	Arthropoda	Insecta	Coleoptera	Cryptophagidae	*Atomaria*	*rubella*
Morph0396	1	W	MN594615; MN594651; MN594687; MN594723; MN594759	MN901899	SAEVG303-20	Leaf Litter	Arthropoda	Insecta	Hemiptera	Lygaeidae	*Nysius*	
Morph0397	1	W*		MN521137	SAEVG304-20	Leaf Litter	Arthropoda	Insecta	Hemiptera	Rhyparochromidae	*Stigmatonotum*	
Morph0398	1	U		MN521138	SAEVG305-20	Leaf Litter	Arthropoda	Arachnida	Araneae	Ctenidae	*Anahita*	
Morph0399	1	U		MN521139	SAEVG306-20	Leaf Litter	Arthropoda	Arachnida	Araneae	Linyphiidae	*Bathyphantes*	*approximatus*
Morph0400	1	U		MN521140	SAEVG307-20	Leaf Litter	Arthropoda	Insecta	Lepidoptera			
Morph0401	1	U		MN521141	SAEVG308-20	Pitfall	Arthropoda	Insecta	Diptera	Phoridae	*Megaselia*	
Morph0402	1	U		MN521142	SAEVG309-20	Pitfall	Arthropoda	Insecta	Diptera	Phoridae	*Megaselia*	
Morph0403	1	U		MN521143	SAEVG310-20	Pitfall	Arthropoda	Insecta	Neuroptera	Mantispidae	*Mantispa*	
Morph0404	1	U		MN521144	SAEVG311-20	Pitfall	Arthropoda	Insecta	Hemiptera	Cicadellidae	*Deltocephalus*	
Morph0406	2	U		MN521145	SAEVG312-20	Pitfall	Arthropoda	Insecta	Hymenoptera	Diapriidae	*Diapriidae*	
Morph0407	1	U		MN521146	SAEVG313-20	Pitfall	Arthropoda	Insecta	Coleoptera	Carabidae	*Scarites*	
Morph0408	3	U		MN521147	SAEVG314-20	Pitfall	Arthropoda	Insecta	Coleoptera	Tenebrionidae	*Gonocephalum*	*pubens*

Table S3: A) ParaFit global and p-value for all datasets calculated using ParaFit program. B) ParaFitLink1 (p.F1) and ParaFitLink2 (p.F2) depicts individual host-symbiont link contributing to co-evolution.

(A)

Dataset	ParaFit global	p-value
**All *Wolbachia***	0.083	0.002
**A supergroup**	0.0010	0.032
**B supergroup**	0.0012	0.191
Cardinium	9.67E-05	0.054
Arsenophonus	6.97E-05	0.097

(B)

**All *Wolbachia***							**A supergroup**							**B supergroup**					
Host	Symbiont	F1. stat	p. F1	F2. stat	p. F2		Host	Symbiont	F1. stat	p. F1	F2. stat	p. F2		Host	Symbiont	F1. stat	p. F1	F2. stat	p. F2
M1	ST-541	7.66E-03	0.009	4.86E-03	0.009		M76	ST-444	1.03E-04	0.214	3.84E-04	0.214		M1	ST-541	5.01E-05	0.252	4.72E-04	0.25
M9	ST-542	7.39E-03	0.012	4.70E-03	0.011		M80	ST-547	-1.74E-05	0.906	-6.51E-05	0.906		M9	ST-542	3.50E-05	0.719	3.30E-04	0.719
M26	ST-543	6.97E-03	0.008	4.42E-03	0.008		M82	ST-548	1.13E-04	0.264	4.22E-04	0.264		M26	ST-543	3.20E-05	0.471	3.01E-04	0.471
M76	ST-444	2.75E-04	0.632	1.75E-04	0.629		M95	ST-550	1.83E-04	0.023	6.85E-04	0.023		M98	ST-541	-2.55E-05	0.901	-2.40E-04	0.901
M80	ST-547	-4.92E-03	0.994	-3.12E-03	0.994		M152	ST-553	3.12E-05	0.573	1.16E-04	0.573		M111	ST-163	4.97E-05	0.318	4.68E-04	0.316
M82	ST-548	5.89E-03	0.018	3.74E-03	0.016		M171	ST-554	1.05E-04	0.147	3.93E-04	0.145		M210	ST-558	5.55E-05	0.131	5.22E-04	0.13
M95	ST-550	1.70E-03	0.29	1.08E-03	0.278		M182	ST-555	1.08E-04	0.121	4.02E-04	0.12		M213	ST-559	4.11E-05	0.547	3.87E-04	0.547
M98	ST-541	-2.53E-03	0.924	-1.61E-03	0.93		M189	ST-556	3.36E-05	0.468	1.25E-04	0.466		M214	ST-560	2.27E-04	0.186	2.14E-03	0.185
M111	ST-163	2.30E-03	0.323	1.46E-03	0.313		M269	ST-562	1.31E-04	0.197	4.89E-04	0.197		M220	ST-559	1.27E-04	0.122	1.20E-03	0.122
M148	ST-552	1.80E-03	0.465	1.14E-03	0.436		M293	ST-564	3.63E-05	0.447	1.35E-04	0.447		M285	ST-41	4.80E-05	0.26	4.52E-04	0.258
M152	ST-553	6.71E-03	0.009	4.26E-03	0.008		M294	ST-565	9.68E-05	0.158	3.61E-04	0.158		M329	ST-566	5.85E-04	0.177	5.51E-03	0.177
M171	ST-554	5.85E-03	0.014	3.71E-03	0.013		M324	ST-575	3.53E-05	0.415	1.32E-04	0.415		M348	ST-568	1.06E-04	0.085	9.94E-04	0.085
M182	ST-555	8.17E-03	0.002	5.19E-03	0.002		M330	ST-567	1.17E-04	0.025	4.37E-04	0.025		M381	ST-572	5.44E-06	0.797	5.12E-05	0.797
M189	ST-556	3.93E-03	0.065	2.50E-03	0.061		M352	ST-569	2.86E-05	0.433	1.07E-04	0.433		M386	ST-573	-3.61E-05	0.915	-3.40E-04	0.915
M206	ST-557	2.59E-03	0.429	1.64E-03	0.39		M375	ST-570	8.87E-05	0.259	3.31E-04	0.258		M396	ST-574	4.40E-05	0.327	4.14E-04	0.327
M210	ST-558	5.96E-03	0.039	3.78E-03	0.034		M376	ST-571	1.82E-04	0.019	6.81E-04	0.019							
M213	ST-559	1.10E-03	0.466	7.01E-04	0.461														
M214	ST-560	2.37E-03	0.104	1.51E-03	0.098		***Cardinium***							***Arsenophonus***					
M220	ST-559	2.99E-03	0.201	1.90E-03	0.192														
M269	ST-562	7.19E-03	0.004	4.57E-03	0.004		Host	Symbiont	F1. stat	p. F1	F2. stat	p. F2		Host	Symbiont	F1. stat	p. F1	F2. stat	p. F2
M285	ST-41	4.54E-03	0.085	2.88E-03	0.077		M45	C45	1.83E-05	0.112	3.59E-04	0.112		M16	A16	3.17E-06	0.283	1.03E-04	0.282
M293	ST-564	8.30E-03	0.001	5.27E-03	0.001		M85	C85	4.81E-05	0.05	9.44E-04	0.05		M70	A70	1.35E-06	0.566	4.36E-05	0.566
M294	ST-565	8.86E-03	0.002	5.62E-03	0.002		M152	C152	4.52E-05	0.039	8.87E-04	0.039		M167	A167	2.94E-06	0.594	9.54E-05	0.594
M324	ST-575	7.39E-03	0.003	4.70E-03	0.002		M171	C171	-2.77E-06	0.812	-5.43E-05	0.812		M260	A260	9.01E-07	0.664	2.92E-05	0.664
M329	ST-566	9.08E-03	0.024	5.77E-03	0.021		M196	C196	5.69E-06	0.505	1.12E-04	0.505		M294	A294	1.05E-05	0.173	3.42E-04	0.173
M330	ST-567	1.32E-05	0.65	8.35E-06	0.648		M248	C248	4.21E-06	0.32	8.26E-05	0.32		M328	A328	6.10E-05	0.045	1.97E-03	0.046

M348	ST-568	3.88E-03	0.162	2.46E-03	0.153		M306	C306	8.42E-06	0.291	1.65E-04	0.29		M329	A329	3.18E-06	0.38	1.03E-04	0.38
M352	ST-569	-1.55E-03	0.859	-9.85E-04	0.866		M328	C328	1.32E-05	0.11	2.59E-04	0.11							
M375	ST-570	-3.25E-03	0.976	-2.06E-03	0.977														
M376	ST-571	1.62E-03	0.29	1.03E-03	0.283														
M381	ST-572	5.63E-03	0.043	3.58E-03	0.041														
M386	ST-573	6.76E-03	0.009	4.30E-03	0.009														
M396	ST-574	5.77E-03	0.043	3.66E-03	0.037														

Table S4: Pairwise genetic distance for all datasets calculated using MEGA7 and represented as percentage. Jukes cantor correction and standard deviation of the mean was obtained from DNAsp v5.10.01.

	**Community Mean (Jukes cantor corrected)**	**Expected mean**	**Database Mean (Jukes cantor corrected)**
Entire *Wolbachia* MLST	8.68 (±0.45)	8.17	8.66 (±0.11)
A supergroup only	2.67*(±0.15)	3.54	3.69 (± 0.26)
B supergroup only	4.17 (±0.66)	3.38	3.43 (±0.10)
Cardinium	1.41* (±0.11)	2.48	2.01 (±0.11)
Arsenophonus	1.19 (±0.26)	1.38	1.55 (±0.11)
